# A novel risk score model based on pyroptosis-related genes for predicting survival and immunogenic landscape in hepatocellular carcinoma

**DOI:** 10.18632/aging.204544

**Published:** 2023-02-24

**Authors:** Hongyu Wang, Bo Zhang, Yanan Shang, Fei Chen, Yumei Fan, Ke Tan

**Affiliations:** 1 Ministry of Education Key Laboratory of Molecular and Cellular Biology, Hebei Key Laboratory of Animal Physiology, Biochemistry and Molecular Biology, College of Life Sciences, Hebei Normal University, Shijiazhuang 050024, Hebei, China

**Keywords:** hepatocellular carcinoma, pyroptosis, molecular subtypes, immunotherapy, drug sensitivity

## Abstract

Background: Hepatocellular carcinoma (HCC) is the third leading cause of cancer worldwide, with high incidence and mortality. Pyroptosis, a form of inflammatory-regulated cell death, is closely associated with oncogenesis.

Methods: Expression profiles of HCC were downloaded from the TCGA database and validated using the ICGC and GEO databases. Consensus clustering analysis was used to determine distinct clusters. The pyroptosis-related genes (PRGs) included in the pyroptosis-related signature were selected by univariate Cox regression and LASSO regression analysis. Kaplan-Meier and receiver operating characteristic (ROC) analyses were performed to estimate the prognostic potential of the model. The characteristics of infiltration of immune cells between different groups of HCC were explored.

Results: Two independent clusters were identified according to PRG expression. Cluster 2 showed upregulated expression, poor prognosis, increased immune cell infiltration and worse immunotherapy response than cluster 1. A prognostic risk signature consisting of five genes (GSDME, NOD1, PLCG1, NLRP6 and NLRC4) was identified. In the high-risk score group, HCC patients showed decreased survival rates. In particular, multiple clinicopathological characteristics and immune cell infiltration were significantly associated with the risk score. Notably, the 5 PRGs in the risk score have been implicated in carcinogenesis, immunological pathways and drug sensitivity.

Conclusions: A prognostic signature comprising five PRGs can be used as a potential prognostic factor for HCC. The PRG-related signature provides an in-depth understanding of the association between pyroptosis and chemotherapy or immunotherapy for HCC patients.

## INTRODUCTION

Liver cancer ranks as the third leading cause of cancer-associated death according to GLOBOCAN 2020 [1]. Hepatocellular carcinoma (HCC), which has seriously affected human health, is the major primary liver cancer [2, 3]. In the past decade, despite great progress in surgery and various treatments, such as radiotherapy, chemotherapy, transarterial chemoembolization (TACE), molecular targeted therapy and minimally invasive surgery, the overall 5-year survival rate is only 18%, and the long-term prognosis of HCC patients still needs to be improved [4–6].

During the process of tumor initiation, development and metastasis, cancer cells gradually form an adaptive tumor immune microenvironment and begin to avoid programmed death and escape immunity. Pyroptosis, a newly identified type of cell death triggered by inflammation, exhibits morphological characteristics of both necrosis and apoptosis [7, 8]. Under physiological conditions, pyroptosis defends against pathogen or bacterial infections. However, excessive pyroptosis tends to lead to sustained amplified inflammatory responses that are involved in various human diseases, such as infectious diseases, cardiovascular diseases, atherosclerosis, diabetic kidney disease, renal ischemia-reperfusion injury and neurodegenerative diseases [9, 10]. Pyroptosis provides new therapeutic strategies for human diseases. More importantly, previous studies have elucidated that pyroptosis is of great significance to tumor progression, and its anti-cancer effects have gradually attracted worldwide attention [11]. Morphologically, the main characteristics of pyroptotic cells include bubble-like protrusions, cellular swelling, and the formation of membrane pores by the gasdermin (GSDM) protein family [12]. The formation of GSDM pores on the plasma membrane eventually leads to cell lysis, releasing many damage-associated molecular patterns (DAMPs), such as ATP, interleukin-1 beta (IL-1β), S100 family proteins, heat shock proteins (HSPs) and high mobility group box protein 1 (HMGB1) [12, 13]. The occurrence of pyroptosis leads to a strong inflammatory response in the body, which then affects the tumor immune microenvironment [14, 15]. Nucleotide-binding domain and leucine-rich repeat-containing receptors (NLRs) and the GSDM family play essential roles in pyroptosis signaling pathways. Noncanonical pathways triggered by caspase 11 in mice and caspase 4/5 in humans and canonical pathways triggered by caspase-1 are generally two modes of pyroptosis [16]. In the canonical pathway, inflammasomes play a role in recruiting apoptosis-associated speck-like protein containing a caspase recruitment domain (ASC) to activate caspase-1, leading to cytokine secretion and GSDMD cleavage [17]. The N-terminus of GSDMD forms pores on the membrane to cause the release of inflammatory factors and cell lysis [13]. Recent studies suggested that caspase-3 could be activated by some stimuli to promote the cleavage of GSDME, leading to pore formation [18]. NLRs, a family of proteins that play a key role in host defense, not only recognize conserved pathogen-associated molecular patterns (PAMPs) but also identify DAMPs [19]. NLRs can induce inflammasome formation [20]. The inflammasome can process signals to trigger a cascade of inflammatory responses. Thus, there are significant associations between NLRs and multiple human diseases related to infection and immunity [21]. NLRs exhibit diverse molecular functions under both physiological and pathological conditions, such as inflammasome assembly, signal transduction, transcription activation and autophagy [22]. Since these novel links between pyroptosis and human diseases may improve our understanding of the pathogenesis of diseases and promote the development of new ways to prevent and treat these diseases, pyroptosis is also receiving widespread attention from clinicians [9, 10]. Recently, numerous studies have demonstrated that inflammasome-regulated pyroptosis is closely interlinked with the pathogenesis of cancer [23]. For example, NLRP6 expression was decreased in gastric cancer and obviously associated with Helicobacter pylori infection, lymph node metastasis, tumor stage and survival rate [24]. Overexpression of NLRP6 reduced cell growth, decreased invasion and migration, and promoted cell apoptosis in gastric cancer cells [24]. Moreover, decreased level of NLRP6 was correlated with unfavorable prognosis in patients with head and neck squamous cell carcinoma (NHSCC), revealing the tumor suppressive role of NLRP6 in gastric cancer and NHSCC [25]. In addition, the protein level of NLRC4 was upregulated and linked with unfavorable prognosis in glioma patients, demonstrating that NLRC4 is a diagnostic biomarker and potential therapeutic target for glioma [26]. Furthermore, loss of NLRC4 impeded colon cancer liver metastasis accompanied by reduced infiltration level of M2 macrophages and IL-1β expression in mice with high-fat diet-triggered nonalcoholic fatty liver disease (NAFLD) [27]. The protein levels of GSDMD were markedly upregulated in non-small cell lung cancer (NSCLC), and upregulated GSDMD was markedly correlated with invasive characteristics and worse prognosis [19, 28]. GSDME protein levels were increased in esophageal squamous cell carcinoma (ESCC) and positively corresponded to a favorable prognosis [29]. Cotreatment with the PLK1 inhibitor BI2536 and cisplatin triggered caspase-3/GSDME axis-dependent pyroptosis in ESCC cells [29]. The high expression of GSDME in tumors can effectively promote the infiltration of different immune cells, and correspondingly, the immune cell infiltration and immune response in GSDME-deficient tumors tend to decrease. This GSDME-dependent pyroptosis, a novel nonapoptotic mechanism of eliminating cancer cells, is downstream of the activated mitochondria-mediated caspase pathway [18, 30]. Nevertheless, the association between pyroptosis-related genes (PRGs) and immunity in HCC remains unclear, and it is vital to construct a new prognostic model of PRGs.

In this study, we performed a comprehensive systematic analysis of PRGs in HCC using TCGA, ICGC and GEO databases. Two independent HCC clusters established by consensus clustering analysis were shown to have different immune cell infiltration and prognostic survival. To further assess the effects of the PRGs in HCC, a five-PRG risk model, including GSDME, NOD1, PLCG1, NLRP6 and NLRC4, was identified to be greatly linked with the overall survival (OS) of HCC patients. We also determined the significance of the signature by exploring the associations between the risk score and immune cell infiltration, clinical features, drug sensitivity and immunotherapy response in HCC patients. These results provide an in-depth understanding of the prognostic power of PRGs and provide immunotherapy strategies and treatments for HCC patients.

## RESULTS

### Expression of pyroptosis-related genes (PRGs) in HCC in the TCGA database

**Figure 1:**
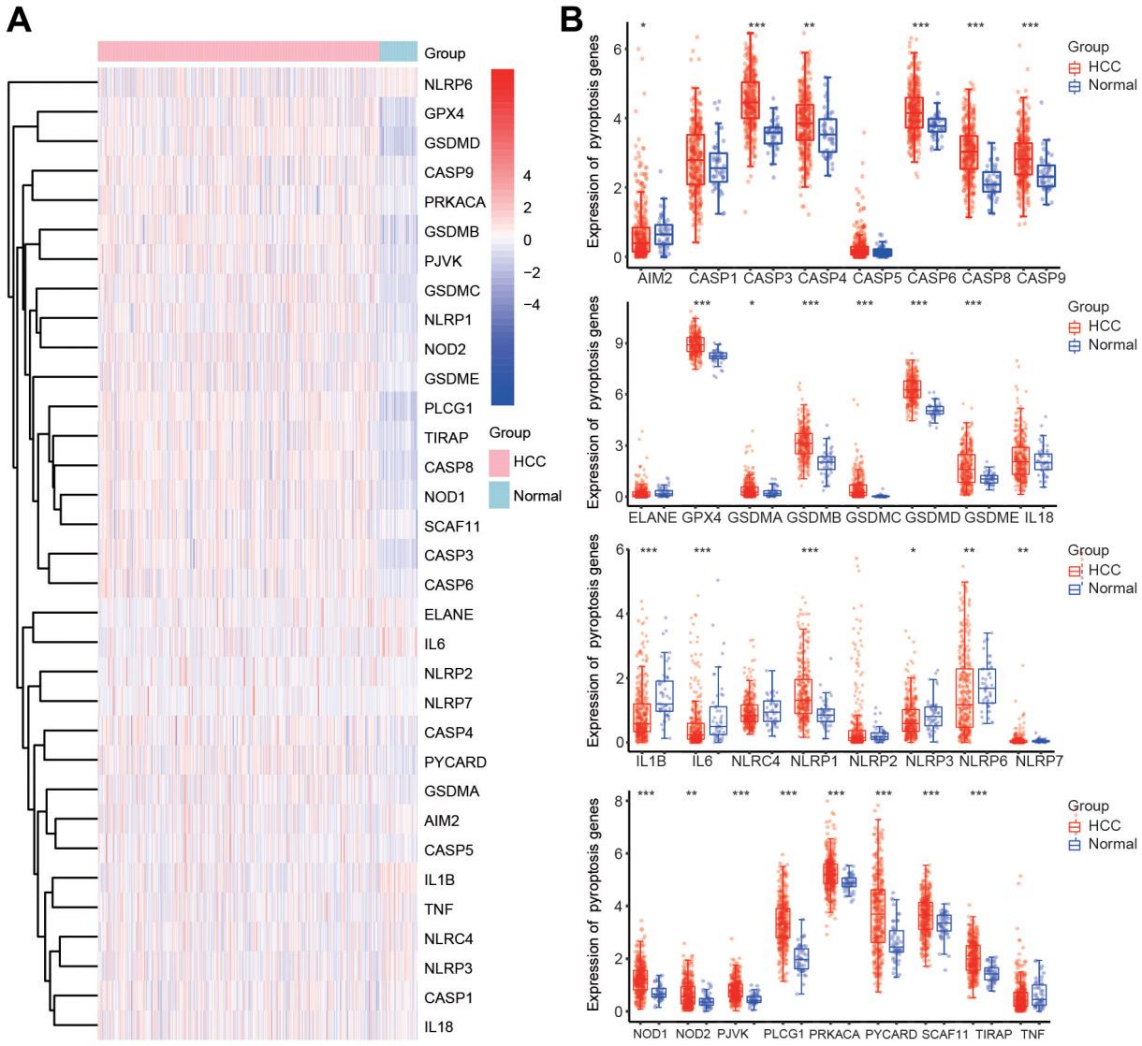
**Differential expression of 33 PRGs in HCC according to the TCGA database.** (**A**) Heatmap of the differential expression of PRGs in HCC samples and normal samples. (**B**) Box diagram of the differential expression of PRGs in HCC samples and normal samples. *p < 0.05, **p < 0.01, ***p < 0.001.

The flow chart depicting the analysis procedure for the present study is shown in Supplementary Figure 1. According to previous studies, we first analyzed the expression of thirty-three PRGs that were of great significance in modulating pyroptotic cell death in HCC. We evaluated the expression of these PRGs to investigate the functions of pyroptosis in HCC using the TCGA database. The expression of most PRGs, including CASP3, CASP4, CASP6, CASP8, CASP9, GPX4, GSDMA, GSDMB, GSDMC, GSDMD, GSDME, NLRP1, NLRP7, NOD1, NOD2, PJVK, PRKACA, PYCARD, PLCG1, SCAF11 and TIRAP, was significantly increased in HCC tissues compared with normal tissues (Figure 1A, 1B). Conversely, markedly decreased expression of AIM2, IL1B, IL6, NLRP3 and NLRP6 was observed in HCC tissues compared with normal tissues (Figure 1A, 1B).

To confirm the altered expression of the thirty-three PRGs, we also investigated the transcriptional level of these PRGs according to the ICGC database. The expression of AIM2, IL18, IL1B, IL6, NLRC4, NLRP2, NLRP3, NLRP6, NLRP7 and TNF was obviously downregulated in HCC tissues (Supplementary Figure 2A, 2B). However, the expression of CASP3, CASP6, CASP8, CASP9, GPX4, GSDMA, GSDMC, GSDMD, GSDME, NLRP1, NOD1, PLCG1, PYCARD and TIRAP was markedly elevated in HCC tissues compared with normal tissues (Supplementary Figure 2A, 2B).

### Construction of an interactive network of PRGs and signaling pathway analysis

**Figure 2:**
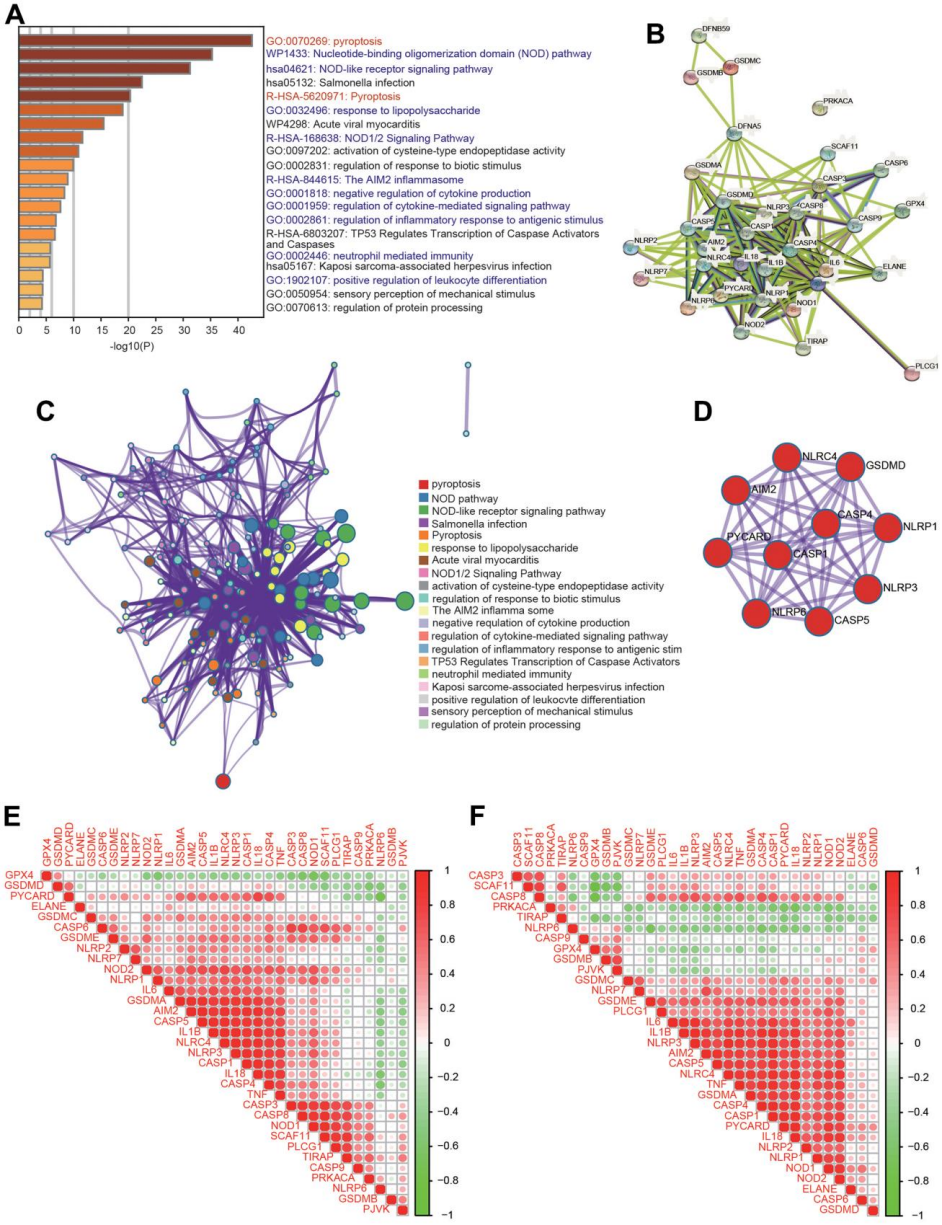
**Functional analysis of 33 PRGs in HCC.** (**A**) The enriched signaling pathways of 33 PRGs were obtained from the Metascape database. (**B**) A PPI network was constructed using the STRING database. (**C**) A gene-gene interactive network was constructed using the Metascape database. (**D**) The hub genes were selected from the PPI network using the Metascape database. (**E**, **F**) Heatmaps demonstrating the correlations among 33 PRGs with Spearman analysis in the TCGA and ICGC databases.

To further investigate the mechanisms of PRGs with differential expression in HCC, signaling pathway analysis was carried out using the Metascape database. Consistent with our speculation, these PRGs were strongly and positively associated with pyroptosis (Figure 2A). Additionally, these PRGs were also remarkably involved in various immunity-related pathways, including the nucleotide-binding oligomerization domain (NOD) pathway, NOD-like receptor signaling pathway, response to lipopolysaccharide, NOD1/2 signaling pathway, AIM2 inflammasome, negative regulation of cytokine production, regulation of cytokine-mediated signaling pathway, regulation of inflammatory response to antigenic stimulus, neutrophil-mediated immunity, and positive regulation of leukocyte differentiation (Figure 2A). Moreover, PPI networks were generated through the Metascape and STRING databases (Figure 2B, 2C). Additionally, the core interactions of PRGs are shown in Figure 2D. We then assessed the correlations among these PRGs. There were positive or negative correlations among the thirty-three PRGs according to the TCGA and ICGC databases (Figure 2E, 2F).

### Consensus clustering analysis of PRGs in HCC

**Figure 3:**
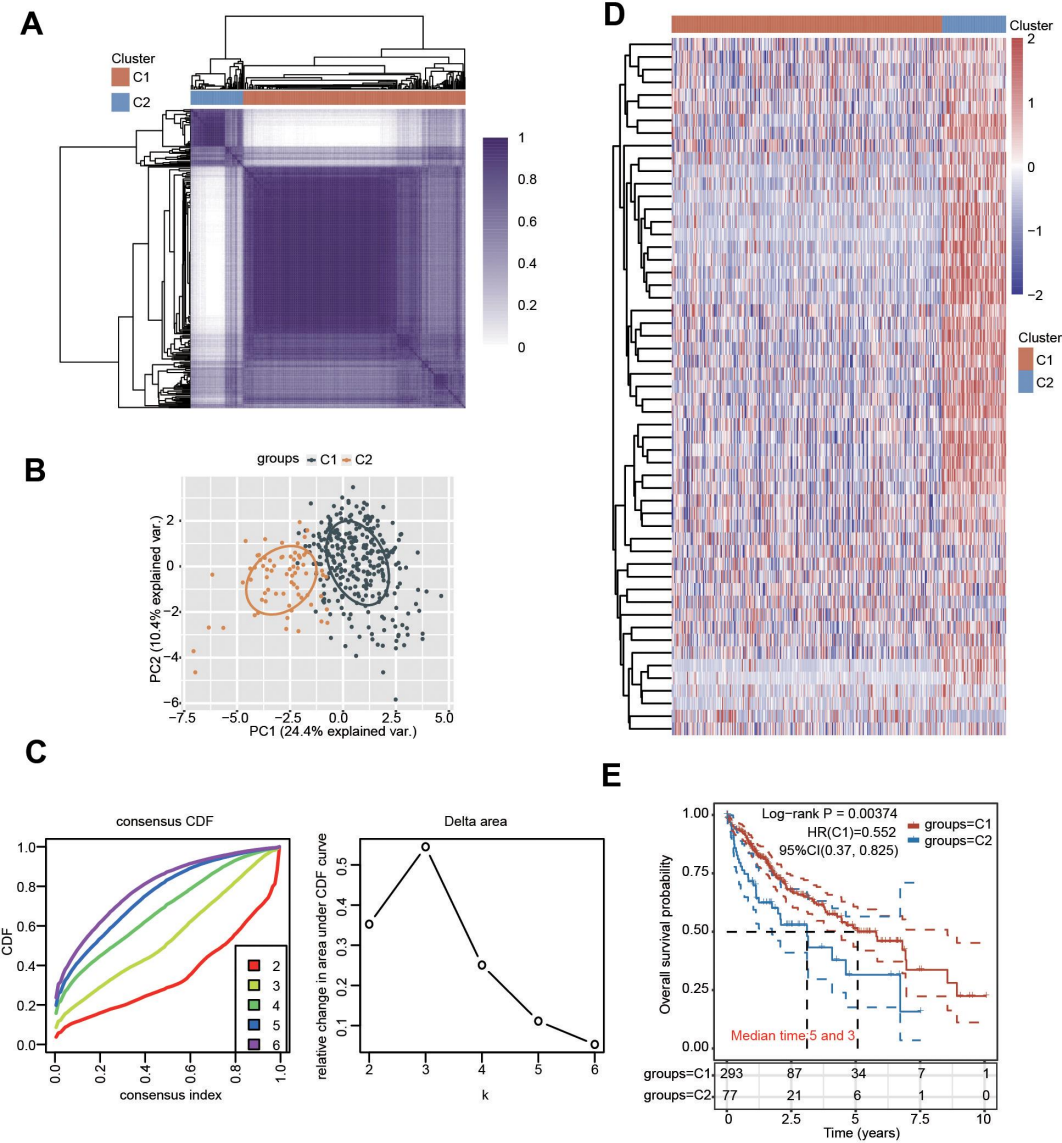
**Identification of distinct clusters of PRGs in HCC.** (**A**, **B**) Two clusters were defined by consensus clustering analysis. (**C**) Cumulative distribution curves for k = 2-6. (**D**) Heatmap showing the expression pattern of PRGs in the two clusters. (**E**) KM analysis showed the OS for the two clusters of HCC patients.

Depending on the diverse expression levels of PRGs, we performed consensus clustering analysis. We identified k = 2 as the variable clustering stability, suitably dividing HCC patients into two subgroups (Figure 3A). Considering the transcriptome data of these 2 clusters, PCA was conducted (Figure 3B, 3C). The expression of these PRGs in the two clusters was further estimated (Figure 3D and Supplementary Figure 3). Most PRGs were highly expressed in cluster 2 (C2) compared with cluster 1 (C1) (Figure 3D and Supplementary Figure 3). Additionally, obvious differences in survival between the two clusters and worse OS were observed in HCC patients in C2 compared with those in C1 (Figure 3E). In addition, there were obvious differences in multiple clinicopathological parameters between the two clusters, including grade, T stage and TNM stage (Table 1).

**Table 1:** Relationships between various clinicopathological characteristics and the two clusters in HCC.

	**Characteristic**	**C1**	**C2**	**P_value**
**Status**	Alive	196	45	
	Dead	97	33	0.168
**Age**	Mean (SD)	59.5 (13.7)	59.3 (12.7)	
	Median [Min, Max]	61 [16,90]	62 [20,85]	0.89
**Sex**	Male	203	47	
	Female	90	31	0.169
**Race**	White	142	42	
	Asian	127	31	
	Black	12	5	
	American Indian	2		0.567
**pT-stage**	T1	157	24	
	T2	63	29	
	T3	34	11	
	T3a	22	7	
	T3b	4	2	
	T4	10	3	
	TX	1		
	T2a		1	
	T2b		1	0.017
**pN-stage**	N0	202	50	
	N1	3	1	
	NX	88	26	0.794
**pM-stage**	M0	214	52	
	M1	4		
	MX	75	26	0.249
**pTNM-stage**	I	147	24	
	II	58	28	
	III	3		
	IIIA	49	16	
	IIIB	5	3	
	IIIC	6	3	
	IV	2		
	IVA	1		
	IVB	2		0.007
**Grade**	G1	51	4	
	G2	141	36	
	G3	89	33	
	G4	7	5	0.008

### Immune cell infiltration in two different clusters

**Figure 4:**
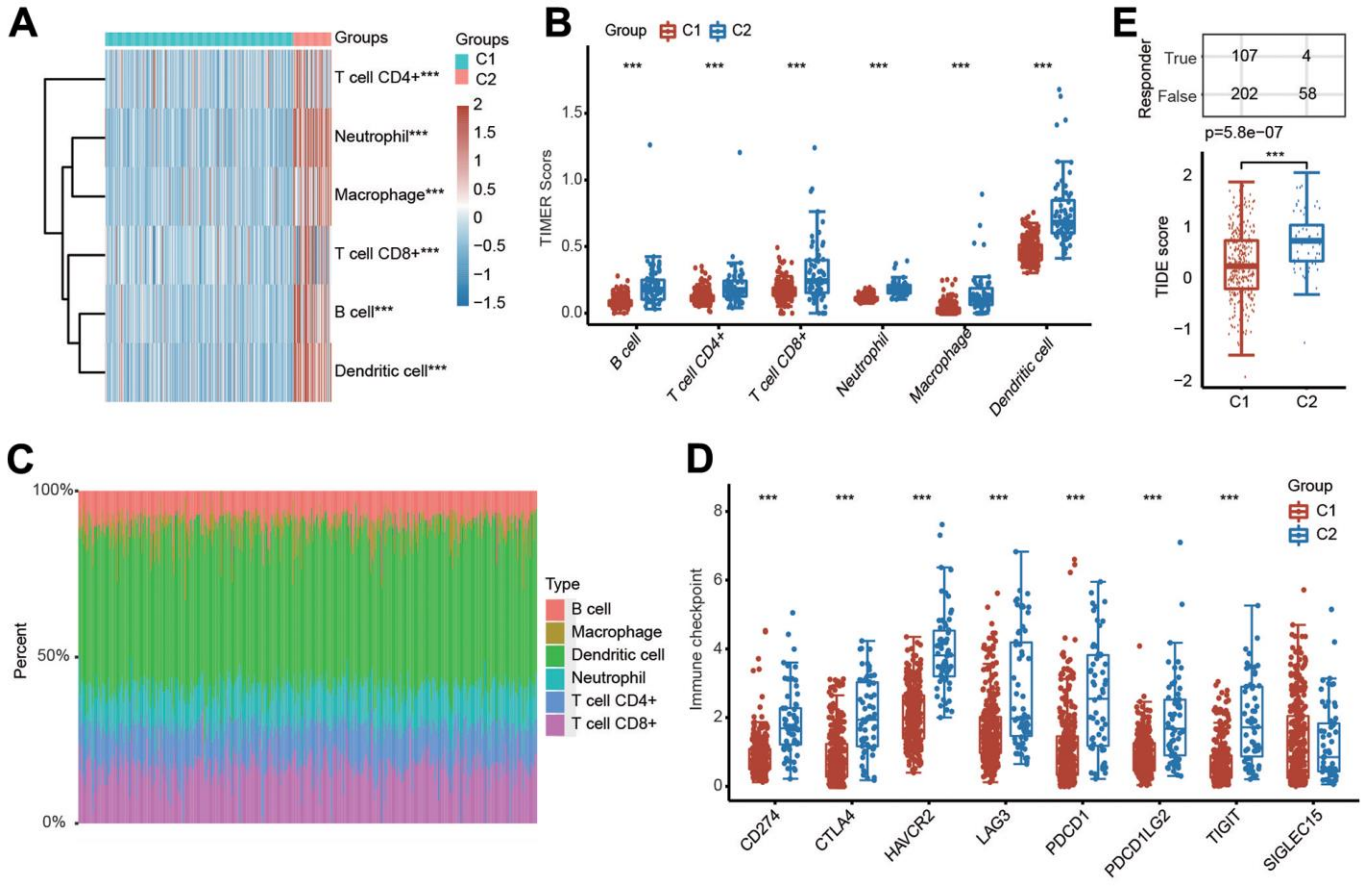
**Evaluation of immune cell infiltration abundance in different clusters of HCC samples by the TIMER algorithm.** (**A**, **B**) Heatmap and box diagram showing the differential infiltration abundance of six types of immune cells in C1 and C2. (**C**) Bar plot demonstrating the composition of a great variety of immune cells in every HCC patient from C1 and C2 analyzed by the TIMER algorithm. (**D**) Box plots indicating the altered expression of immune checkpoint genes in C1 and C2. (**E**) Box plots showing the TIDE scores in the two clusters. ***p < 0.001.

Because PRGs were closely associated with the immune response (Figure 2A), we then explored the relationship between different clusters and the tumor immune microenvironment. The results of the TIMER algorithm illuminated that the infiltration scores of six major immune cells, including CD4+ T cells, B cells, CD8+ T cells, macrophages, neutrophils and dendritic cells, in C1 were obviously lower than those in C2 (Figure 4A, 4B). The percentage abundance of infiltrated immune cells in each HCC patient is shown with different colors and immune cell types (Figure 4C). We also investigated the influence of different clusters on the expression levels of well-known important immune checkpoint genes and observed that the expressions of CD274, PDCD1, PDCD1LG2, CTLA4, LAG3, HAVCR2 and TIGIT were markedly downregulated in C1 compared with C2 (Figure 4D). More importantly, the TIDE score was lower in C1 than in C2, suggesting a better response to immunotherapy in C1 (Figure 4E).

Furthermore, the relationships between patient clusters and immune cell infiltration were also confirmed by the CIBERSORT algorithm. The infiltration abundances of activated CD4+ memory T cells, resting memory CD4+ T cells, regulatory T cells (Tregs), M0 macrophages, resting NK cells, activated mast cells, naïve B cells, memory B cells, neutrophils and resting mast cells in C1 and C2 were obvious different (Supplementary Figure 4A, 4B). The percentage abundance of infiltrated immune cells in each HCC patient is indicated with different colors and immune cell types according to the CIBERSORT algorithm (Supplementary Figure 4C).

### Molecular and functional enrichment analyses of the differences in two clusters of PRGs

**Figure 5:**
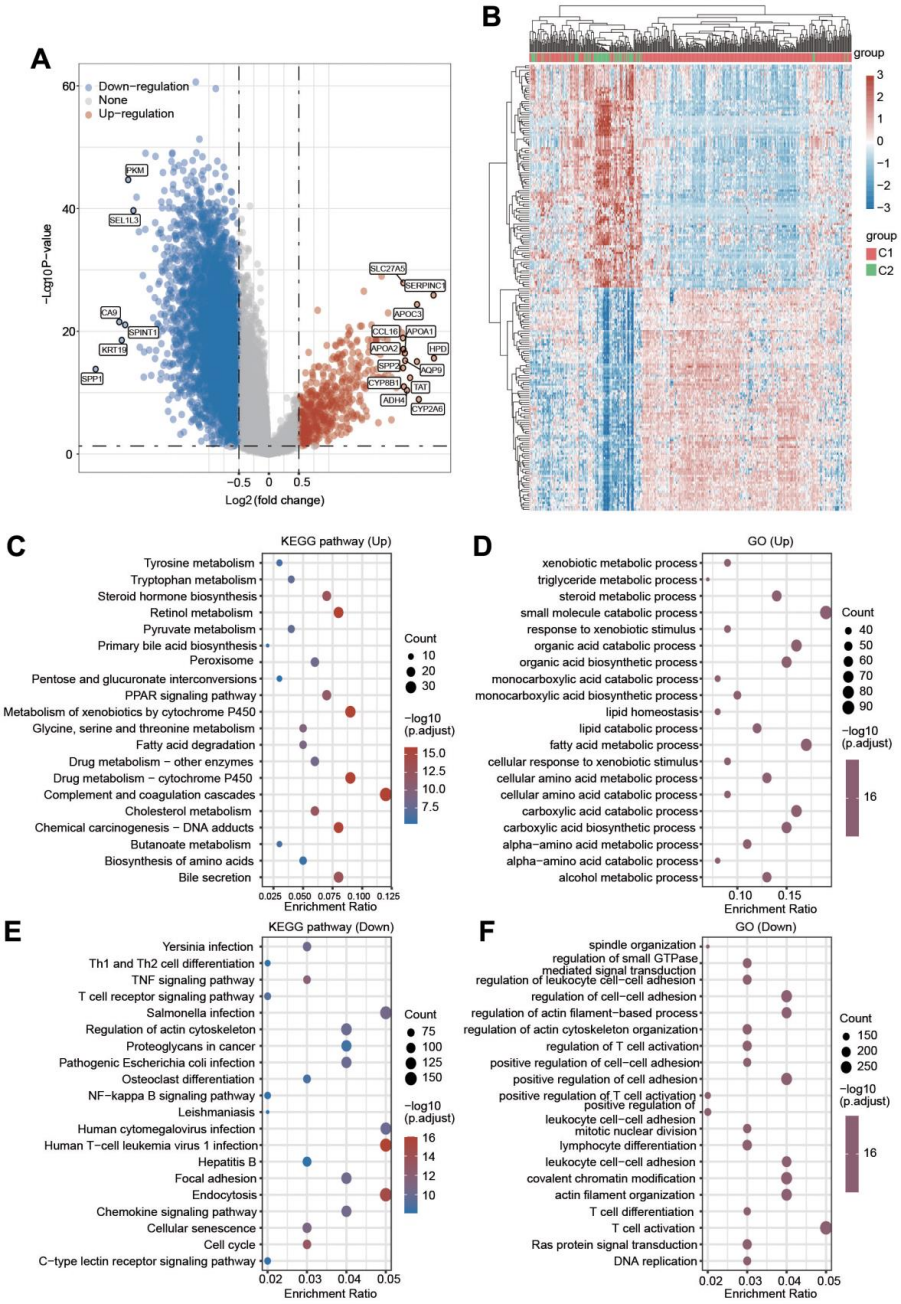
(**A**) Volcano plot displaying the upregulated and downregulated genes in C2 compared with C1. (**B**) A clustering heatmap showing the changed expression of genes in two clusters after the deep filtration of genes with p < 0.05 and |log2 (fold change)|> 1.5 as thresholds. (**C**, **D**) KEGG and GO analyses were applied to explore the different signaling pathways for the upregulated genes. (**E**, **F**) KEGG and GO analyses were applied to explore the different signaling pathways for the downregulated genes.

To further elucidate the molecular mechanism underlying the difference between C1 and C2, we then investigated the alteration of gene expression between these two clusters. As shown in Figure 5A, 5B, 486 genes were significantly upregulated and 6643 genes were downregulated in C1 compared with C2. Next, GO and KEGG analyses were performed to explore the different signaling pathways between C1 and C2 using upregulated or downregulated genes. The top 5 enriched KEGG pathways for upregulated genes were complement and coagulation cascades, metabolism of xenobiotics by cytochrome P450, drug metabolism-cytochrome P450, retinol metabolism and bile secretion (Figure 5C). The top 5 enriched pathways for upregulated genes were small molecule catabolic process, fatty acid metabolic process, carboxylic acid catabolic process, organic acid catabolic process and carboxylic acid biosynthetic process (Figure 5D). The top 5 enriched KEGG pathways for downregulated genes were endocytosis, Salmonella infection, human cytomegalovirus infection, human T-cell leukemia virus 1 infection and chemokine signaling pathway (Figure 5E). Additionally, the top 5 enriched GO terms for downregulated gene pathways were T-cell activation, covalent chromatin modification, regulation of cell–cell adhesion, actin filament organization, and positive regulation of cell adhesion (Figure 5F). These data imply that the difference between C1 and C2 is linked with metabolism- and immunity-associated signaling pathways.

### Genetic mutation and drug sensitivity prediction of the two clusters in HCC

**Figure 6:**
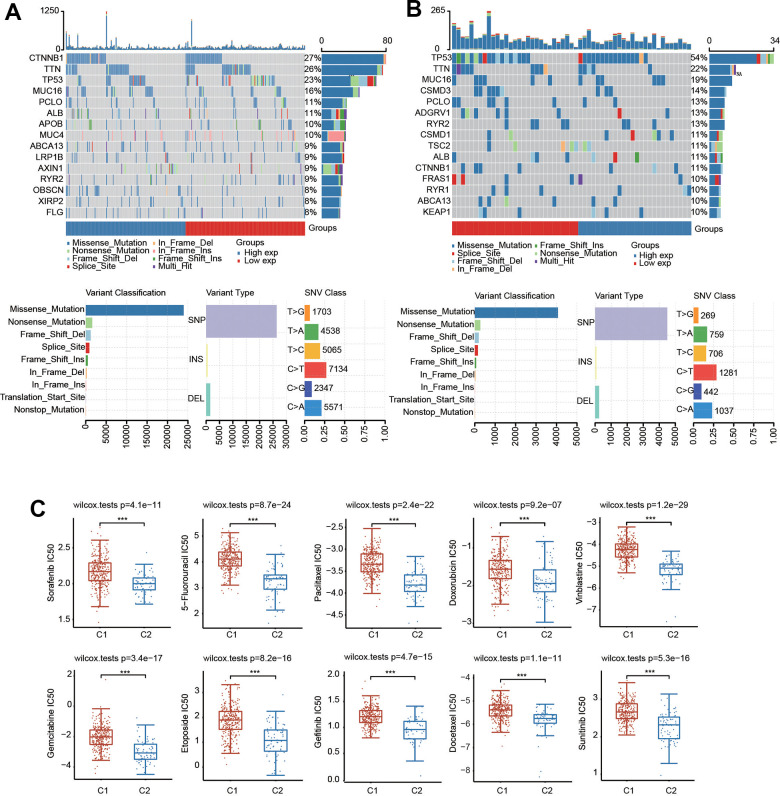
**Mutational landscape and drug sensitivity of two clusters.** (**A**) The landscape of mutation profiles in C1. (**B**) The landscape of mutation profiles in C2. Variant classification, variant types and SNV classification are shown. (**C**) Comparison of drug sensitivity in the two clusters. ***p < 0.001.

We then generated the mutation profiles of HCC patients in C1 and C2 using the TCGA database. In C1, the top five genes with high mutation rates were CTNNB1, TTN, TP53, MUC16 and PCLO (Figure 6A). In contrast, TP53, TTN, MUC16, CSMD3 and PCLO were the most common mutation cohorts of genes altered in C2 (Figure 6B). In addition, missense was the primary type of mutation, and SNP was the major variant in both C1 and C2 (Figure 6A, 6B). The results of SNV class revealed that the most common type of the two risk groups was C > T (Figure 6A, 6B).

We also evaluated the difference in sensitivity to chemotherapeutic drugs in these two clusters. There was a significant difference in the IC50 values of sorafenib, sunitinib, paclitaxel, gefitinib, etoposide, 5-fluorouracil, docetaxel, doxorubicin, vinblastine and gemcitabine between the two clusters, suggesting that C1 was more resistant to these drugs (Figure 6C).

### Identification and construction of a PRG-related prognostic signature in the TCGA database

**Figure 7:**
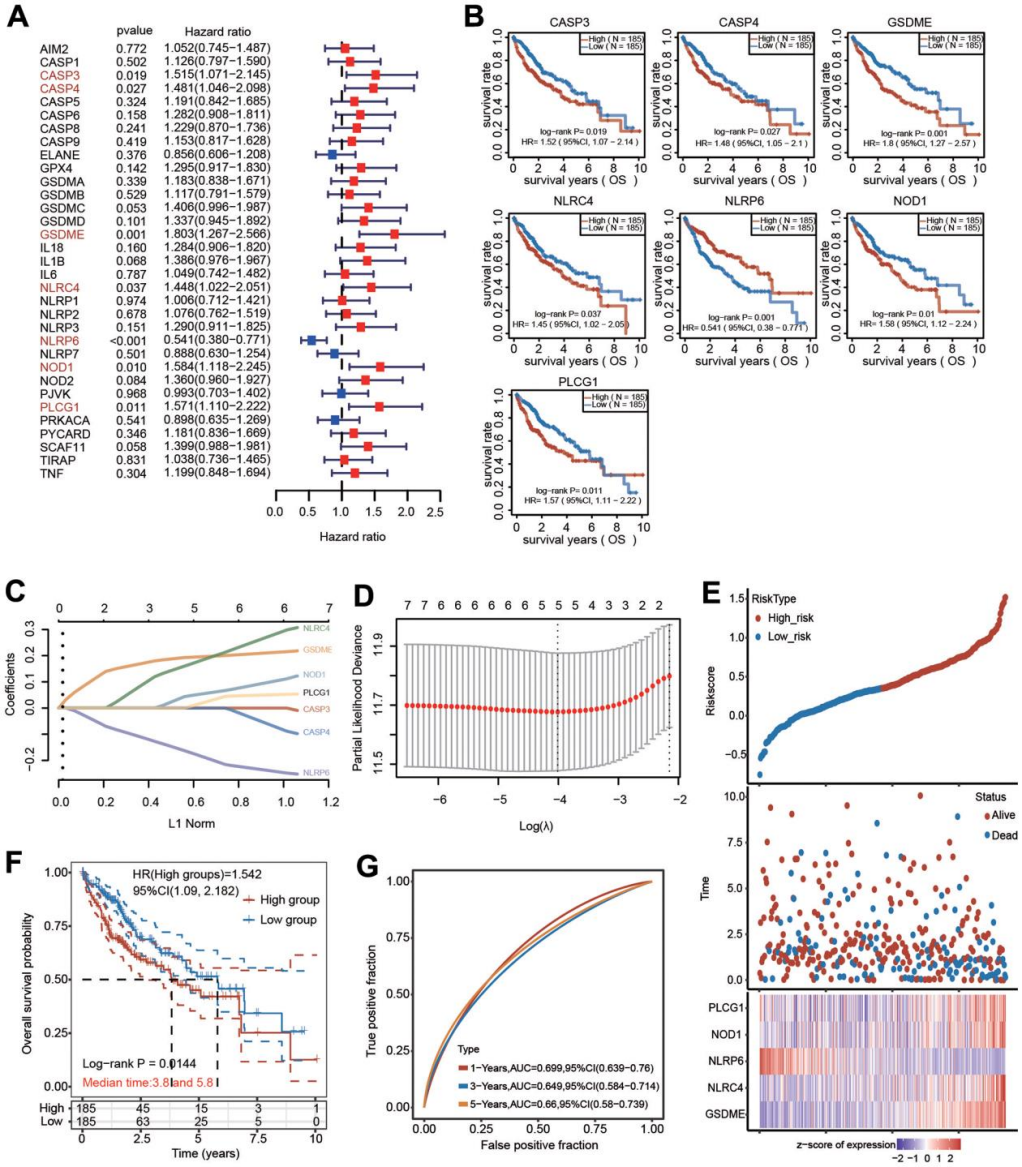
**Construction of a five-PRG signature model in the TCGA-HCC cohort.** (**A**) Forest plot of univariate Cox regression to select the genes with prognostic potential. (**B**) KM analysis revealed the prognostic value of CASP3, CASP4, GSDME, NLRC4, NLRP6, NOD1 and PLCG1 with the log-rank test. (**C**, **D**) A prognostic model containing 5 PRGs was built using LASSO Cox regression analysis. (**E**) The risk score and OS status of each case. (**F**) KM analysis for OS between the low-risk group and high-risk group. (**G**) The AUC of time-dependent ROC curves was shown.

We carried out univariate Cox regression analysis of 33 pyroptosis regulators to select PRGs with prognostic value. Seven hub genes, including CASP3, CASP4, GSDME, NLRC4, NLRP6, NOD1 and PLCG1, were selected with a cutoff of p < 0.05 (Figure 7A). Consistently, the altered expression of CASP3, CASP4, GSDME, NLRC4, NLRP6, NOD1 and PLCG1 also corresponded with favorable or unfavorable OS in HCC patients according to the KM analysis (Figure 7B). These seven candidate genes were considered as prognostic factors in HCC.

LASSO Cox regression analysis based on the optimum λ value was then performed to build a prognostic model for the candidate PRGs to address collinearity. The risk score was calculated as follows: risk score = (0.1974 × GSDME) + (0.1926 × NLRC4) + (-0.1947 × NLRP6) + (0.0581 × NOD1) + (0.0272 × PLCG1) (Figure 7C, 7D). The risk score, survival outcome and 5 PRG gene expression of each HCC patient in TCGA database are vividly shown (Figure 7E). Of note, KM curve analysis results revealed that HCC patients in the high-risk group had unfavorable OS compared with those in the low-risk group (Figure 7F). ROC analysis was applied to verify the sensitivity and specificity of the prognostic model. The areas under the ROC curve (AUCs) were 0.699 for 1-year survival, 0.649 for 3-year survival and 0.66 for 5-year survival (Figure 7G).

### Confirmation of the PRG-related prognostic signature in the ICGC database

A similar calculation was applied to the data from the ICGC database to verify the availability of the prognostic signature. The risk score was calculated as follows: risk score = (0.2485 × GSDME) + (-0.5484 × NLRC4) + (-0.9524 × NLRP6) + (0.1684 × NOD1) + (-0.3558 × PLCG1) (Supplementary Figure 5). We also divided the HCC patients into high-risk and low-risk subgroups in the ICGC database. HCC patients with high risk had a reduced survival time and a higher risk of mortality (Supplementary Figure 5A). The HCC patients in the high-risk subgroup had poor prognosis compared with those in the low-risk subgroup based on KM analysis, indicating good accuracy of this prognostic signature (Supplementary Figure 5B). The AUC values were 0.635 for 1-year survival, 0.687 for 2-year survival and 0.724 for 3-year survival (Supplementary Figure 5C). Collectively, these data suggested that the pyroptosis-related prognostic signature model could distinguish favorable prognoses in HCC patients.

### Independent prognostic potential of the PRG signature according to various clinicopathological parameters

**Figure 8:**
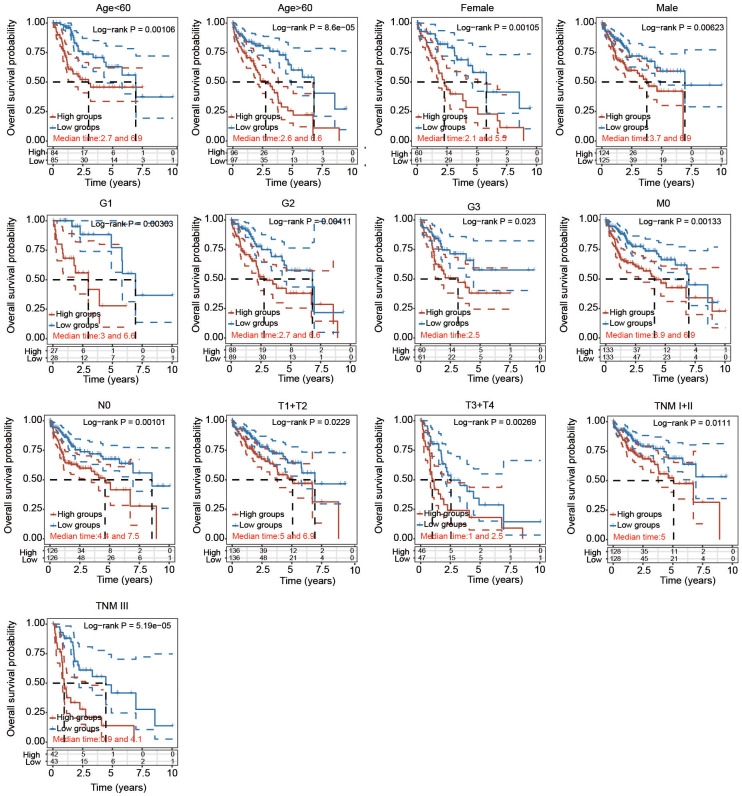
**Prognostic potential of the risk score with different clinical parameters.** KM analysis of OS between two subgroups stratified by age < 60, age > 60, male, female, T1 + T2, T3 + T4, G1, G2, G3, M0, N0, TNM I+II and TNM III with the log-rank test according to the TCGA database.

To further certify the prognostic value of the 5-PRG signature, the association between various clinicopathological parameters and risk score was explored. The high-risk score of the 5-PRG signature was obviously associated with worse OS in young (< 60 years), old (> 60 years), female, male, early stage (T1 + T2), advanced stage (T3 + T4), early grade (G1 and G2), advanced grade (G3), M0, N0, TNM stage I+II and TNM stage III HCC patients (Figure 8). Together, these data suggest that the 5-FRG signature can predict OS among each stratum of age, sex, stage and grade and further prove the good stratification ability of the 5-PRG prognostic model.

### Univariate and multivariate Cox regression analyses and construction of the nomogram

**Figure 9:**
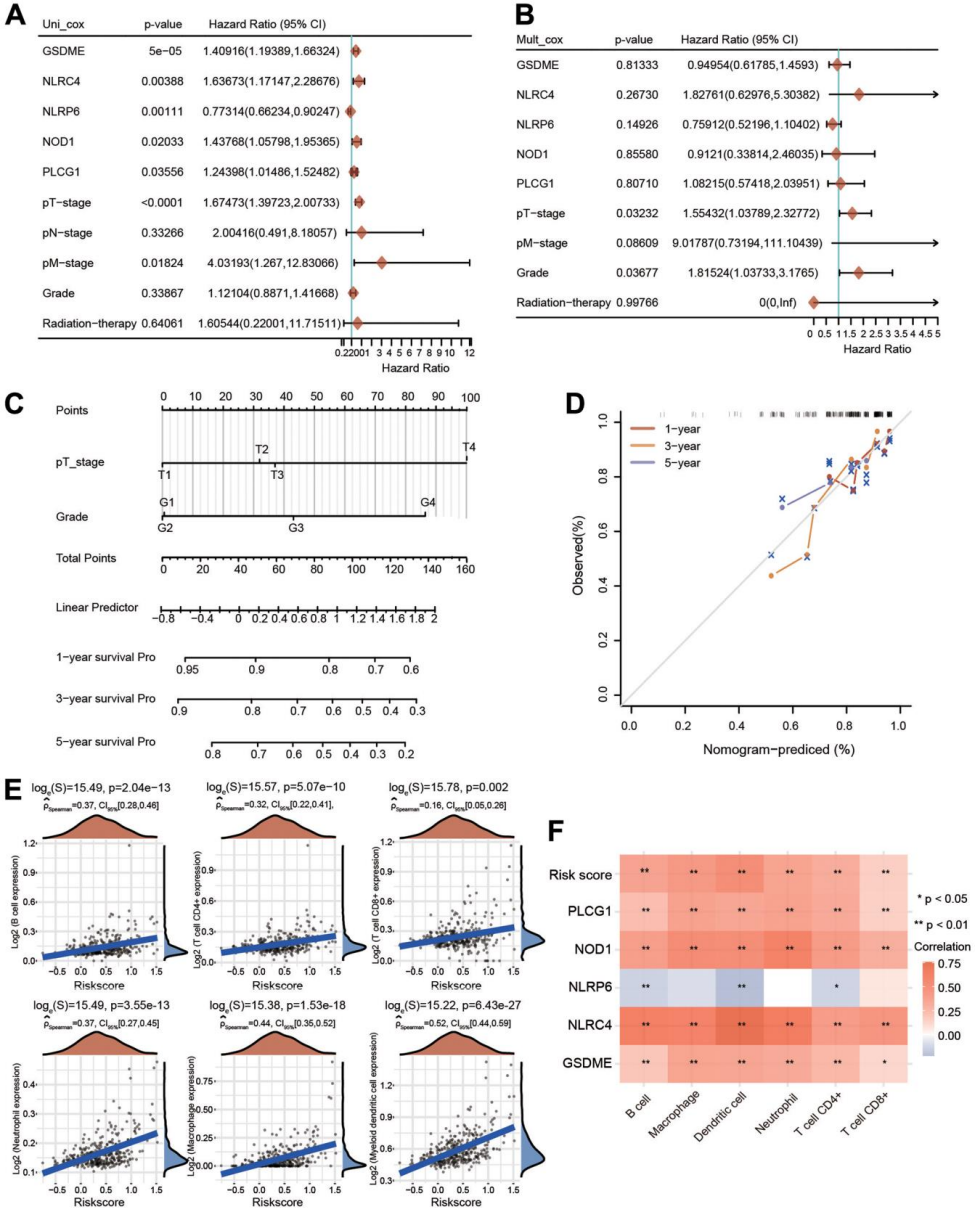
**Univariate and multivariate Cox regression analyses for risk score and construction of a nomogram.** (**A**, **B**) Univariate Cox regression and multivariate Cox regression analyses of five PRGs and clinical features. (**C**) A nomogram containing the prognostic signature and different clinicopathological parameters was constructed. (**D**) Calibration curve of the actual 1-, 3-, and 5-year OS. (**E**) Association between the risk score and the infiltration abundances of six immune cells. (**F**) Heatmap depicting the correlations between the risk score and five PRGs and the infiltrated abundances of six types of immune cells. *p < 0.05, **p < 0.01.

To further assess the prognostic value of the PRG-related prognostic signature in HCC patients, we performed univariate and multivariate Cox regression analyses (Figure 9A, 9B). Following univariate Cox regression analysis, GSDME, NLRC4, NLRP6, NOD1, PLCG1, T stage and M stage were clearly related to OS (Figure 9A). Following the results of multivariate Cox regression analysis, T stage and grade had obvious correlations with OS (Figure 9B). A nomogram model integrating T stage and grade was further constructed to predict the OS of HCC patients based on multivariate regression analysis (Figure 9C). The calibration plots of the nomogram illuminated good agreement between the nomogram-predicted and actual 1-, 3- and 5-year survival rates (Figure 9D).

### Immune cell infiltration analysis of the risk model

We then estimated the relationship between the immune cell infiltration and the risk score in HCC. The infiltrated levels of six major immune cell types were investigated utilizing the TIMER method. The risk score was strongly linked with the infiltrated levels of B cells, neutrophils, macrophages, CD4+ T cells, CD8+ T cells and dendritic cells (Figure 9E). In addition to the risk score, GSDME, NOD1, PLCG1 and NLRC4 were also significantly positively correlated with the infiltration abundances of these immune cells, whereas NLRP6 was negatively linked with the infiltrated abundances of B cells, CD4+ T cells and dendritic cells (Figure 9F).

### Expression of 5 hub PRGs

**Figure 10:**
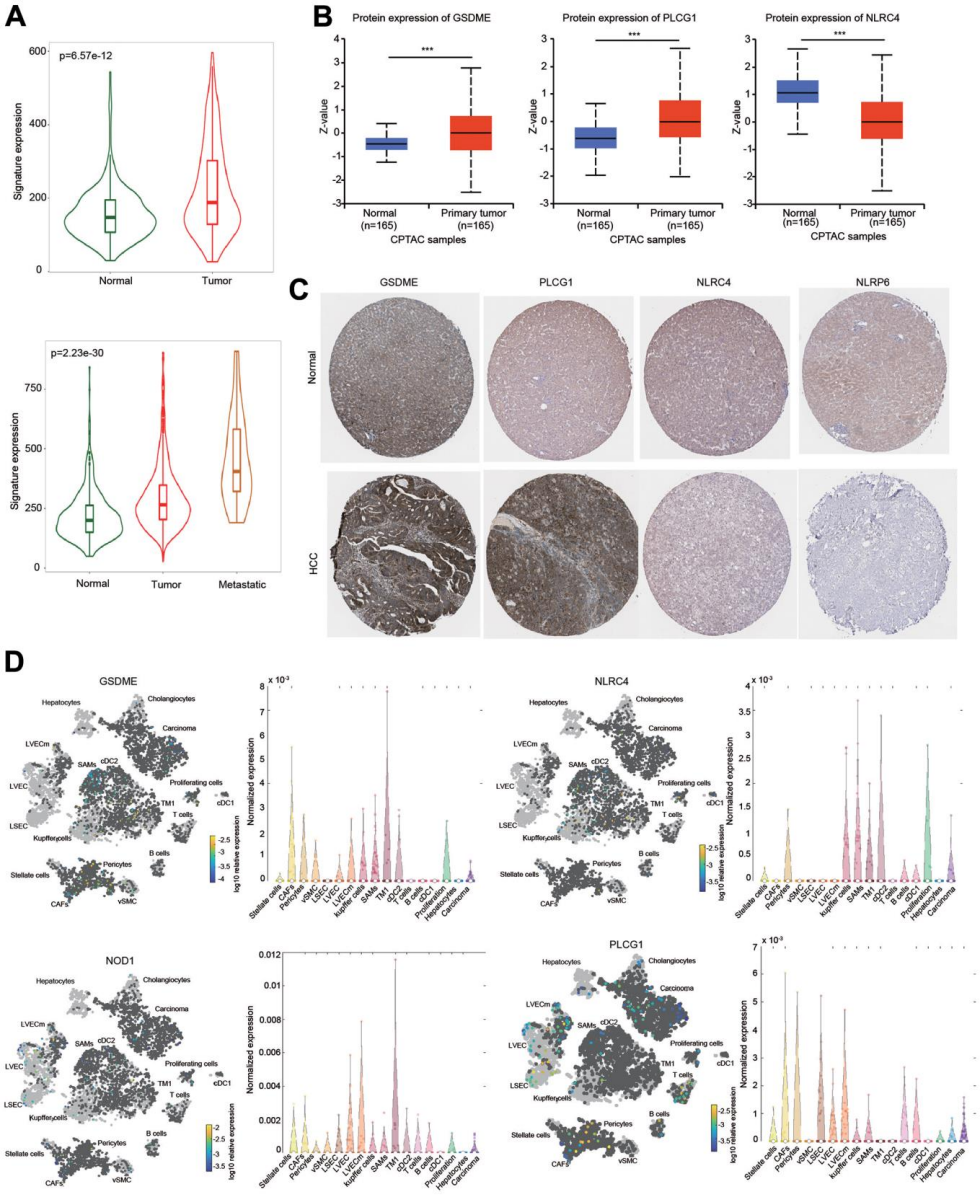
**Expression of the prognostic signature and PRGs in HCC samples and normal liver samples.** (**A**) The expression of the prognostic signature in HCC was examined using the TMNplot database. (**B**) The protein levels of GSDME, NLRC4 and PLCG1 were examined using the CPTAC database. (**C**) IHC analysis of the protein levels of GSDME, PLCG1, NLRC4 and NLRP6 using the HPA database. (**D**) Differential expression and distribution of GSDME, PLCG1, NLRC4 and NOD1 in HCC based on single-cell RNA-sequence analysis using the Human Liver Browser database. ***p < 0.001.

The expression of the five-gene signature was obviously elevated in HCC tissues compared with normal tissues (Figure 10A). The expression of this signature was much higher in metastatic tissues (Figure 10A). Next, the transcriptional levels of these five hub genes were separately examined based on the HCCDB database. Increased expression of GSDME and PLCG1, and decreased expression of NLRC4 were found in HCC tissues in most GEO datasets (Supplementary Figure 6). Moreover, NOD1 expression was increased and NLRP6 expression was decreased in three different datasets (Supplementary Figure 6).

The protein expression level of these genes was examined according to the CPTAC database. The protein levels of GSDME and PLCG1 were higher, while the protein level of NLRC4 was lower in HCC than in normal tissues (Figure 10B). IHC results were obtained from the HPA database to further estimate the protein expression levels of GSDME, NLRC4, PLCG1 and NLRP6. The protein levels of GSDME and PLCG1 were upregulated in HCC, which was consistent with the CPTAC data. In contrast, the protein levels of NLRC4 and NLRP6 were downregulated in HCC compared with normal liver tissues (Figure 10C).

We further examined these gene expressions using single-cell RNA-sequence data. Elevated expression levels of GSDME, NOD1 and PLCG1 in HCC tissues were observed (Figure 10D). Interestingly, these four PRGs were expressed not only in liver cancer cells but also in some immune cells, which may be one reason for the immune cell infiltration of the risk score (Figure 10D).

### Genetic mutation and drug sensitivity of 5 PRGs in HCC

We then explored the genetic mutation profiles of these five PRGs using the cBioPortal online tool. The PLCG1 gene had the highest mutation frequency (2%), followed by GSDME (1.1%), NLRP6 (1.1%), NLRC4 (1.1%) and NOD1 (0.3%) (Supplementary Figure 7A, 7B). The mutation was the primary type for these 5 genes (Supplementary Figure 7B). We also investigated the correlations between the five PRGs and several tumorigenesis-associated pathways, including the cell cycle, apoptosis, DNA damage response, EMT, hormone ER, hormone AR, RAS/MAPK, PI3K/AKT, RTK and TSC/mTOR pathways. The 5 hub PRGs were essentially linked with the inhibition or activation of these signaling pathways (Supplementary Figure 7C).

**Figure 11:**
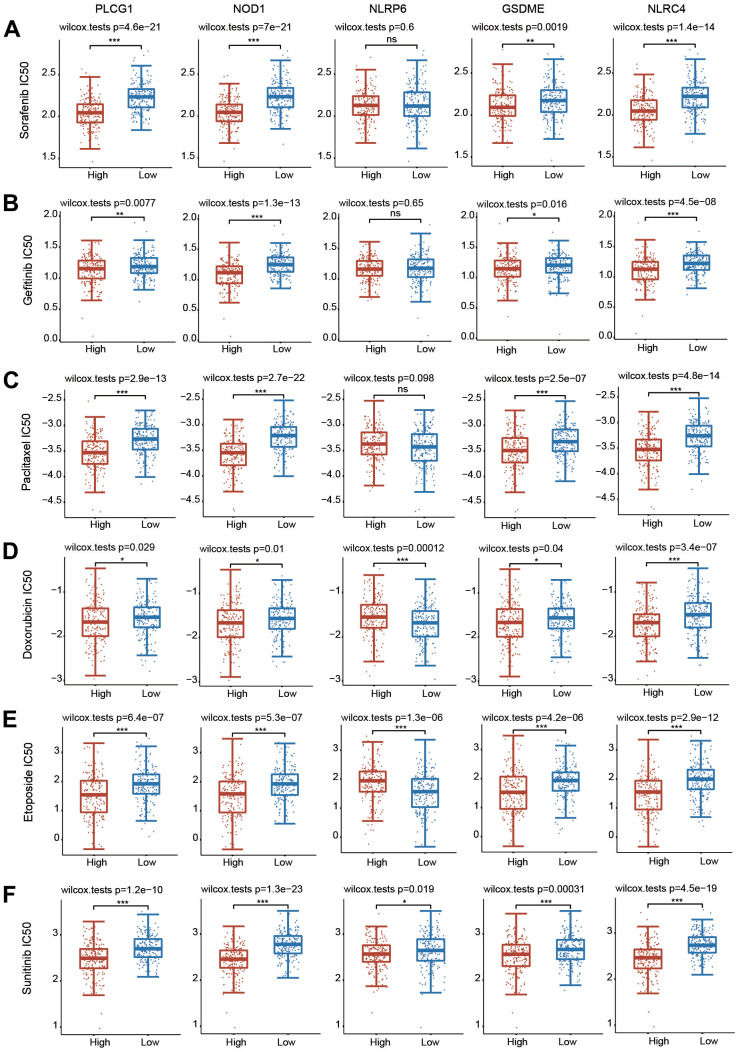
**Correlations between the 5 PRGs and drug sensitivity in HCC.** (**A**–**F**) The relationships between the expression of 5 PRGs and drug sensitivity were explored based on the GDSC database through the pRRophetic package. *p < 0.05, **p < 0.01, ***p < 0.001.

We then evaluated whether the 5 PRGs affected the sensitivity of chemotherapy drugs using the GDSC database (Figure 11). According to the median expression of these 5 PRGs, HCC patients were separated into high-expression and low-expression groups. The IC50 values of all these chemotherapeutic drugs were significantly different between the high-expression group and the low-expression group (Figure 11). These data illustrate that HCC patients with increased expression of 5 PRGs are more sensitive to common chemotherapeutic agents.

## DISCUSSION

Liver cancer is the third leading cause of cancer-related death in the world, and its high incidence rate and mortality seriously threaten human health [1]. The HCC patients do not have obvious diagnostic symptoms at the early stage, so the opportunity for surgery may have been lost at the time of diagnosis, and the survival rate of HCC patients is still unsatisfactory [3]. Thus, it is urgent to deeply and systematically understand the molecular mechanism and open up new diagnostic strategies and treatment methods for HCC. Pyroptosis is a new form of regulated cell death (RCD) that causes cell membrane rupture and death via continuous cell expansion, resulting in the release of cell contents, which in turn activates a strong inflammatory response [11]. Compared with apoptosis, pyroptosis occurs faster and is accompanied by the release of a large number of proinflammatory factors, leading to the rapid death of cancer cells [31]. A growing number of studies have illuminated that pyroptosis-related molecules play a role in promoting tumor development and provide a new idea for the treatment of liver cancer [32–34].

In this study, two independent clusters were identified using consensus clustering analysis according to the expression levels of 33 PRGs. Importantly, PRGs in C2 had increased expression, and patients in C2 exhibited a worse prognosis than those in C1. Meanwhile, essential differences in terms of grade, T stage and TNM stage between C1 and C2 were observed (Table 1). C2 was enriched in immunity-related biological pathways and strongly correlated with prognosis and immune infiltration. In addition, to obtain a novel prognostic signature to predict OS, we selected pyroptosis regulators related to prognosis in HCC. Based on the prognostic potential of PRGs in HCC patients, we established and validated the risk prediction models of five PRGs (GSDME, NLRC4, NLRP6, NOD1 and PLCG1) and separated the HCC patients into a high-risk group and a low-risk group. The univariate and multivariate Cox regression analysis results illustrated that the established PRG risk model was an independent prognostic model for HCC patients. Combined with the established clinicopathological characteristics, ROC analysis proved that the risk model had more benefits in predicting the OS of patients with HCC. To expand our risk model, we further established a novel nomogram model to predict OS. At the same time, the actual OS is highly consistent with the model predictions as the results of the calibration curve.

Chemotherapy and immunotherapy are important adjunct treatments for patients with HCC [4]. The development of new anticancer drugs is a time-consuming, high-investment and high-risk project. Often, the birth of new anticancer drugs requires several years or even decades of research, development and validation. Sorafenib, a multitargeted tumor drug, can selectively target the receptors of certain signaling pathways to facilitate apoptosis, suppress angiogenesis and inhibit cancer cell proliferation [35]. Sorafenib is an effective first-line therapy for late-stage HCC [36, 37]. Although sorafenib is less toxic and well tolerated, it still has some special adverse effects, which should be considered in clinical research and application. Moreover, according to clinical observations, the overall effective rate of treatment for liver cancer is relatively low. Additionally, sorafenib resistance is becoming more common. Fortunately, many other broad-spectrum anticancer drugs, including 5-fluorouracil, docetaxel, doxorubicin, etoposide, gefitinib, gemcitabine, paclitaxel, vinblastine and sunitinib, are also used as treatment strategies for liver cancer patients. In the current study, according to the GDSC database, the relationships between clusters and chemotherapeutic drug sensitivity were investigated. The sensitivity of the two clusters to common chemotherapeutic drugs was obviously different, and cluster 2 HCC patients may benefit from these drugs. In addition, the risk model containing five PRGs was also significantly correlated with sensitivity to these drugs. In the high PRG expression group, the IC50 value of chemotherapeutic agents was obviously decreased, indicating that HCC patients with elevated PRG expression may gain more therapeutic benefits from these drugs through pyroptosis, which may make the treatment of HCC more effective and have fewer side effects.

As an inflammatory type of RCD, pyroptosis was identified by cell swelling, membrane rupture and pore formation, leading to the release of intracellular contents, including IL-1β and IL-18, and ultimately causing a cascade-amplified inflammatory action [7]. The essential components of pyroptosis, including inflammasomes, GSDM proteins and cytokines, are all associated with the development, invasion and metastasis of tumors [15]. Cleavage of GSDM family members, such as GSDMD and GSDME, mediated by cysteine proteases is the key process that causes pyroptosis [38]. Previous studies have shown that GSDME is downregulated in some human cancers and might act as a tumor suppressor [39, 40]. The DNA methylase inhibitor decitabine (5-aza-2'-deoxycytosine) could downregulate the expression of GSDME, thereby preventing the proliferation and colony formation ability of melanoma, gastric cancer and CRC cells, and may reduce the invasive ability of breast cancer cells [41]. In addition, GSDME is associated with etoposide resistance [42]. Loss of GSDME facilitates the resistance of melanoma cell lines to etoposide, which can be rescued by overexpression of GSDME [42]. Treatment of lung cancer cells with inhibitors of KRAS, EGFR or ALK results in caspase-3-regulated activation of GSDME, thereby increasing the anticancer efficacy of these drugs [43, 44]. In mouse tumor models, knockdown of GSDME enhanced tumor growth, whereas ectopic expression of GSDME inhibited tumor growth [45]. Importantly, the tumor inhibitory effect of GSDME was dominated by killing cytotoxic lymphocytes, as this effect was markedly abolished in mice with loss of perforin or in mice deficient in CD8+ T and NK cells [45]. CAR-T cells induce pyroptosis by sequentially releasing granzyme B, activating caspase-3 and cleaving GSDME [46]. Pyroptosis-associated factors in turn activate caspase-1 in macrophages, leading to cleavage of GSDMD, which ultimately induces cytokine release syndrome [46]. Consistently, knockout of the GMEDE gene in B16 melanoma greatly decreased the survival rate in tumor-implanted mice. Therefore, GSDM genes not only trigger pyroptosis in tumor cells but also activate antitumor immunity [44, 47]. NOD1, a member of the pattern recognition receptor (PRR) family, is involved in various pathologies, especially cancer. NOD1 is expressed in some types of cells, including endothelial cells, hematopoietic cells and various immune cells (e.g., neutrophils, macrophages, monocytes, NK cells, and lymphocytes) [48]. These findings are consistent with our observations. NOD1 activation elicits antigen-specific T-cell immune responses primarily through Th2 polarization [49]. Additionally, NOD1 stimulates Th1, Th2 and Th17 immune responses along with other innate immune TLRs [49]. Additionally, NOD1 activation also contributes to the B-cell antigen receptor-assisted survival of mature B cells [50]. Activation of NOD1 also promoted chemokine production and specific recruitment of neutrophils in mice [51]. A recent study demonstrated that activation of NOD1 facilitated oncogenesis by promoting autophagy-dependent macrophage reprogramming and triggering myeloid-derived suppressor cell (MDSC) expansion and immunosuppressive ability through arginase-1 activity in colorectal cancer [52]. In contrast, NOD1 expression was markedly decreased in HCC tissues, and overexpression of NOD1 greatly prevented tumorigenesis and increased the response to chemotherapeutic drugs through suppression of the SRC/MAPK pathway *in vitro* and *in vivo* [53]. These results imply that NOD1 exerts its tumor-suppressive effect on HCC. In the current study, we observed that NOD1 expression was markedly increased in HCC tissues in some datasets and that NOD1 was also expressed in immune cells through single-cell RNA sequence analysis. PLCG1, a primary subtype of phospholipase C (PLC), is directly activated by diverse membrane receptors. Upon T-cell activation, as a phospholipase, PLCG1 can cleave phosphatidylinositol 4,5-diphosphate in the plasma membrane into two second messengers: inositol 1,4,5 triphosphate and diacylglycerol. Inositol 1,4,5-triphosphate causes calcium release from the endoplasmic reticulum, increases the intracellular calcium concentration and activates NFAT, while diacylglycerol activates specific isoforms of protein kinase C (PKC) [54, 55]. Recent bioinformatics analysis identified that PLCG1 was frequently highly expressed and mutated in various cancers and was involved in tumorigenesis as an oncogene [56]. Elevated expression of PLCG1 was linked with poor survival and tumor progression in lower-grade glioma (LGG) patients [57]. Knockdown of PLCG1 significantly reduced the proliferation, migration and invasiveness of IDH wild-type LGG cells [57]. The PLCG1-mediated signaling pathway also regulated tumor metastasis. The PLCG1/PKCθ axis accelerated STAT3 activation and promoted the proliferation and survival of cutaneous T-cell lymphoma cells [55]. These results have highlighted the important role of these PRGs in immunity and oncogenesis.

A growing body of research has revealed that the five core prognostic PRGs are also closely related to various human diseases. GSDME expression was elevated in the renal tubules of patients with systemic lupus erythematosus (SLE) and pristane-induced lupus mice. Knockout of GSDME significantly alleviated SLE pathogenesis by suppressing GSDME-regulated pyroptosis of renal cells [58]. These data suggest that GSDME-mediated pyroptosis is involved in the pathogenesis of SLE and that GSDME may be a potential therapeutic target for SLE. Loss of GSDME effectively ameliorated cisplatin- or ischemia–reperfusion-induced inflammation and acute kidney injury by inhibiting caspase-3/GSDME-induced pyroptosis [59]. In fact, some chemotherapeutic drugs, including cisplatin and doxorubicin, can trigger GSDME cleavage in human renal cells. Knockdown of GSDME attenuated doxorubicin- or cisplatin-triggered cell pyroptosis [60]. Therefore, GSDME-modulated pyroptosis may play a vital role in chemotherapy-induced nephrotoxicity. Moreover, loss of GSDME also aggravated skin damage in UVB-treated mice by promoting the infiltration and activation of neutrophils [61]. Previous studies have identified NOD1 as a key player in host-microbial defense and multiple inflammatory diseases. There is a direct link between NOD1 and atherosclerosis. *In vivo* experiments indicated that deficiency of NOD1 reduced the risk of atherosclerotic thrombosis by inhibiting leukocyte infiltration and decreasing macrophage apoptosis [62]. NOD1 expression was upregulated in the adipose tissue of patients with metabolic syndrome or gestational diabetes [63, 64]. Interestingly, the polymorphism in NOD1 (Glu266Lys) was associated with dietary saturated fat and insulin sensitivity in humans aged 20-29 years [65]. Whole body or hematopoietic depletion of NOD1 significantly decreased high-fat diet (HFD)-associated glucose and insulin resistance in mice [66, 67]. Another study indicated that, loss of NOD1 accelerated obesity in mice fed a HFD, accompanied by increased levels of free thyroidal T4, reduced expression of uncoupling protein 1 (UCP1) in brown adipose tissues, and enhanced infiltration of inflammatory cells in white adipose tissues and liver tissues, suggesting a protective role of NOD1 against inflammation and obesity [68]. Infection with Japanese encephalitis virus (JEV) greatly elevated the transcriptional and protein expression of NOD1 in mice, and knockout of NOD1 enhanced resistance to JEV infection by inhibiting the neuroinflammatory response and multiple downstream signaling pathways [69]. Knockout of NOD1 also significantly decreased the number of isolated lymphoid follicles in the distal ileum and colon of mice and greatly increased the total number of bacteria in the ileum to affect intestinal homeostasis [70]. Compared with wild-type (WT) mice, mice lacking NOD1 are more likely to be infected with early pneumococcal septicemia, which implies that NOD1 plays a key role in initiating innate defense and promoting a rapid response to infection [71]. These results imply that the physiological function of NOD1 in the intestine is crucial to maintain the homeostasis between the microbiota and host immune system. PLCG1 is a vital regulator of cellular signaling. In mice, specific knockout of PLCG1 in neural progenitor cells resulted in axonal guidance defects in the dorsal midbrain during embryogenesis. Moreover, in adult PLCG1-deficient mice, structural changes in the corpus callosum, olfactory tubercle, and substantia innominate were observed. These data indicated that PLCG1 may play key roles in the development of white matter structure by regulating the netrin-1/deleted in colorectal cancer (DCC) complex signaling pathway [72]. Mice with GABAergic neuron-specific deletion of PLCG1 showed handling-induced recurrent seizures with a reduced number of GABAergic synapses, decreased hippocampal inhibitory synaptic transmission, anxiety alleviation and fear memory disorder [73]. Numerous studies have extensively investigated the immune functions of NLRC4 in response to bacterial infection. For example, mice with NLRC4 deficiency had low resistance to *Salmonella Typhimurium* and *Legionella pneumophila* infections and exhibited elevated bacterial burden [74]. When mice were infected with *Shigella*, intestinal mucosa thickening, shrinkage of the cecum, macroscopic edema, and acute weight loss were observed in NLRC4-/- mice, suggesting that NLRC4 conferred resistance to *Shigella* infection [75]. In NLRC4 knockout mice, bacterial flagellin, one of the main innate immune activators in the intestine, failed to induce the expression of IL-18 and IL-1β, indicating that NLRC4 was necessary to rapidly generate inflammasome cytokines [76]. Lack of NLRC4 also aggravated dextran sulfate sodium (DSS)-induced acute colitis and increased flagellate-caused mortality in mice [76]. Recently, increasing evidence has revealed the important functions of NLRP6 in microbial infection-associated inflammation. Mice lacking NLRP6 were highly resistant to infection with a variety of bacterial pathogens, such as *Salmonella typhimurium*, *Listeria monocytogenes* and *Escherichia coli*. When NLRP6-deficient mice were infected with these bacterial pathogens, the number of circulating monocytes and neutrophils increased, accompanied by activation of the mitogen-activated protein kinase (MAPK) and nuclear factor-κB (NF-κB) signaling pathways. In contrast, NLRP6-/- mice showed increased parasite shedding and significant susceptibility to *Cryptosporidium* infection compared with WT control mice [77]. NLRP6 knockout mice exhibited spontaneous intestinal hyperplasia, large recruitment of inflammatory cells, and deterioration of DSS-induced colitis. The lack of NLRP6 in mouse colon epithelial cells led to a decrease in IL-18 levels and a change in fecal microbiota composition. Compared with WT controls, NLRP6-/- mice infected with encephalomyocarditis virus or murine norovirus 1 had increased mortality and viremia [78]. Mechanistically, NLRP6 bound to viral RNA in cooperation with Asp-Glu-Ala-His (DEAH) box helicase 15 (DHX15) to induce the expression of interferons and interferon-stimulated genes [78]. Additionally, the expression of NLRP6 was increased in intestinal tissues when mice were infected with *Candida albicans*. The colonization of *Candida albicans* facilitated HCC growth in WT mice, but this effect disappeared in NLRP6-/- mice, suggesting that NLRP6 could promote the occurrence and development of HCC [79]. Although there are some contradictory experimental results, NLRP6 undoubtedly participates in the regulation of innate immunity [80].

Immunotherapy aims to activate the human immune system to kill tumor cells and inhibit tumor growth. The targets of immunotherapy are not tumor cells and tissues but the human body's own immune system [81, 82]. The increased expression of immune checkpoint molecules on cancer cells and/or tumor-infiltrating immune cells can inhibit antitumor immunity. Previous studies have confirmed the clinical efficacy of the application of PD-1 or PD-L1 in inhibiting the progression of advanced HCC [83, 84]. Thus, immunotherapy has become a novel treatment approach representing an effective and promising option against HCC. In our current research, both TIMER and CIBERSORT analyses demonstrated that the two clusters exhibited different infiltrated abundances of various immune cells. Interestingly, C2 exhibited higher immune cell infiltration and immune checkpoint gene expression. Moreover, the risk score of the 5-PRG signature was also markedly and positively linked with the infiltrated abundances of six major immune cells. Moreover, the single-cell RNA sequencing analysis results indicated that the core PRGs in the prognostic signature were expressed in both liver cancer cells and different immune cells. More importantly, we also observed that patients in C2 corresponded to higher TIDE scores according to the TIDE algorithm, indicating a worse response to immunotherapy. In summary, the identified distinct clusters and prognostic signature play a critical role in mediating immune cell infiltration and immunotherapy response.

Despite the promising findings obtained, our study still has several limitations. First, a pyroptosis-related prognostic model was constructed by using retrospective data from different databases to predict the survival rate of HCC patients. More large-scale data are needed to assess the application potential of the five PRG-based risk score models. Second, the expression of PRGs in different databases is not consistent. Most HCC patients in the TCGA-HCC database were Caucasian, and it is not clear whether the expression of PRGs and the prognostic signature has a similar tendency in other races and datasets. Third, the molecular functions of the five PRGs identified in this study need to be verified by more in-depth *in vitro* and *in vivo* experiments and clinical data to further explore their roles and their impact on immune cell infiltration and immunotherapy in HCC.

In summary, our analysis results provided insight into the expression pattern of the PRGs and constructed a risk score model and nomogram for prognosis prediction. The two independent clusters and the 5-PRG risk score, which integrated pyroptosis and immunological features with GSDME, NOD1, PLCG1, NLRP6 and NLRC4, could reliably predict prognosis and immunotherapy response in HCC patients. Additionally, the risk score-based nomogram model has promising clinical applications.

## MATERIALS AND METHODS

### Data collection and process

The mRNA expression data and relevant clinical information for patients with HCC (371 HCC samples and 50 normal samples) were downloaded from the TCGA database. RNA sequencing (RNA-seq) data from the ICGC (International Cancer Gene Consortium) database, containing 202 normal samples and 240 HCC samples, were downloaded and used as the validation cohort. Moreover, expression of 5 PRGs in different GEO datasets were downloaded from the HCCDB database [36, 85].

### Identification of differentially expressed PRGs

PRGs were collected from a previous study [86]. The expression profiles of 33 PRGs were directly downloaded from TCGA and ICGC databases. The “ggplot2” and “pheatmap” packages of R language were used to identify differentially expressed PRGs with a P value <0.05. The online STRING (https://string-db.org/) and Metascape (https://metascape.org/gp/index.html#/main/step1) platforms were used to construct the gene-gene interaction and protein-protein interaction (PPI) networks.

### Consensus clustering analysis of PRGs

The PRGs were subjected to unsupervised clustering analysis with the R package “ConsensusClusterPlus”. Principal component analysis (PCA) was carried out to estimate the gene expression patterns among different clusters. Clustering heatmaps were generated using the “pheatmap” package. Kaplan-Meier (KM) analysis was performed to reveal the difference in survival among different clusters by using the “survival” and “survminer” packages.

### Construction of the risk score

Univariate regression analysis was first applied to select PRGs that were correlated with prognosis in HCC. Then, LASSO (least absolute shrinkage and selection operator) regression analysis with the R package “glmnet” was applied to construct the risk score model after univariate regression analysis. The equation was established as follows: risk score = sum of coefficients × prognostic PRG expression levels. KM curves and receiver operating characteristic (ROC) curves were further utilized to examine the prognostic ability of the risk model.

### Construction of the nomogram

Univariate Cox regression and multivariate Cox regression analyses were applied to verify whether the risk model was linked with prognosis in HCC. In addition, a nomogram was constructed based on age, sex, tumor (T), node (N), metastasis (M) and risk score using the R package “rms”.

### Mutation landscapes in two clusters

Tumor mutation burden (TMB) could predict the response to some different forms of immunotherapy and across multiple types of cancer. The mutation landscapes of the two clusters were visualized and compared through the R package “maftools”.

### Analysis of differentially expressed genes

The R package “limma” was utilized to acquire the differentially expressed genes between different clusters with |log2 (fold change)|> 1.5 and p < 0.05. Additionally, Kyoto Encyclopedia of Genes and Genomes (KEGG) pathway enrichment and Gene Ontology (GO) analyses were performed to explore the potential function of differentially expressed genes using the “ClusterProfiler” package. Heatmaps and boxplots were generated using the “pheatmap” and “ggplot2” packages, respectively.

### Immune cell infiltration abundance in HCC

The infiltration abundances of a variety of immune cells between the two risk groups were investigated using the CIBERSORT and TIMER algorithms. The infiltrated abundance of various immune cells in every HCC sample was explored using the “immunedeconv” package. The heatmap results are shown by the R package “pheatmap”.

### Associations between clusters and immunotherapy response

The expression levels of major immune checkpoint genes between cluster 1 and cluster 2 were compared to show the difference under immunotherapy between the two subgroups. Additionally, the responses to immunotherapy were assessed with the TIDE (tumor immune dysfunction and exclusion) algorithm using the R packages “ggplot2” and “ggpubr”. The TIDE score of HCC was obtained from http://tide.dfci.harvard.edu.

### Immunohistochemistry analysis

Immunohistochemical (IHC) staining results were directly obtained from the HPA (Human Protein Atlas) database (https://www.proteinatlas.org/) as described previously [36, 87]. The protein levels of PRGs in normal liver tissues and HCC tissues were compared through IHC staining.

### Targeted therapy drug prediction

The chemotherapeutic response for each sample was predicted according to the largest publicly available Genomics of Drug Sensitivity in Cancer (GDSC) database (https://www.cancerrxgene.org/) with the R package “pRRophetic”. The IC50 (half-maximal inhibitory concentration) was assessed through ridge regression.

### Statistical analysis

All statistics were performed using R software (version 4.0.3). The Wilcoxon test was used for comparisons between two different subgroups. In KM analysis, the log-rank test was applied to estimate the difference in survival rate between subgroups. A p value less than 0.05 was set as statistically significant for all analyses.

### Data availability

The datasets analyzed for this study can be downloaded from the TCGA database (https://portal.gdc.cancer.gov), ICGC database (https://dcc.icgc.org/releases/current/Projects) and HCCDB database (http://lifeome.net/database/hccdb/home.html). All data generated or analyzed during this study are included in this article and its Supplementary Material files. Further inquiries can be directed to the corresponding authors.

### Consent for publication

All authors approved the manuscript for publication.

## Supplementary Material

Supplementary Figures
