# Supplementary material for: A novel risk score model based on pyroptosis-related genes for predicting survival and immunogenic landscape in hepatocellular carcinoma
Source: Aging (Albany NY). 2023 Feb 24;15(5):1412–44. doi: 10.18632/aging.204544 (PMC10042690; doi:10.18632/aging.204544)
Supplement: Supplementary Figures [file aging-15-204544-s001.pdf]

SUPPLEMENTARY FIGURES

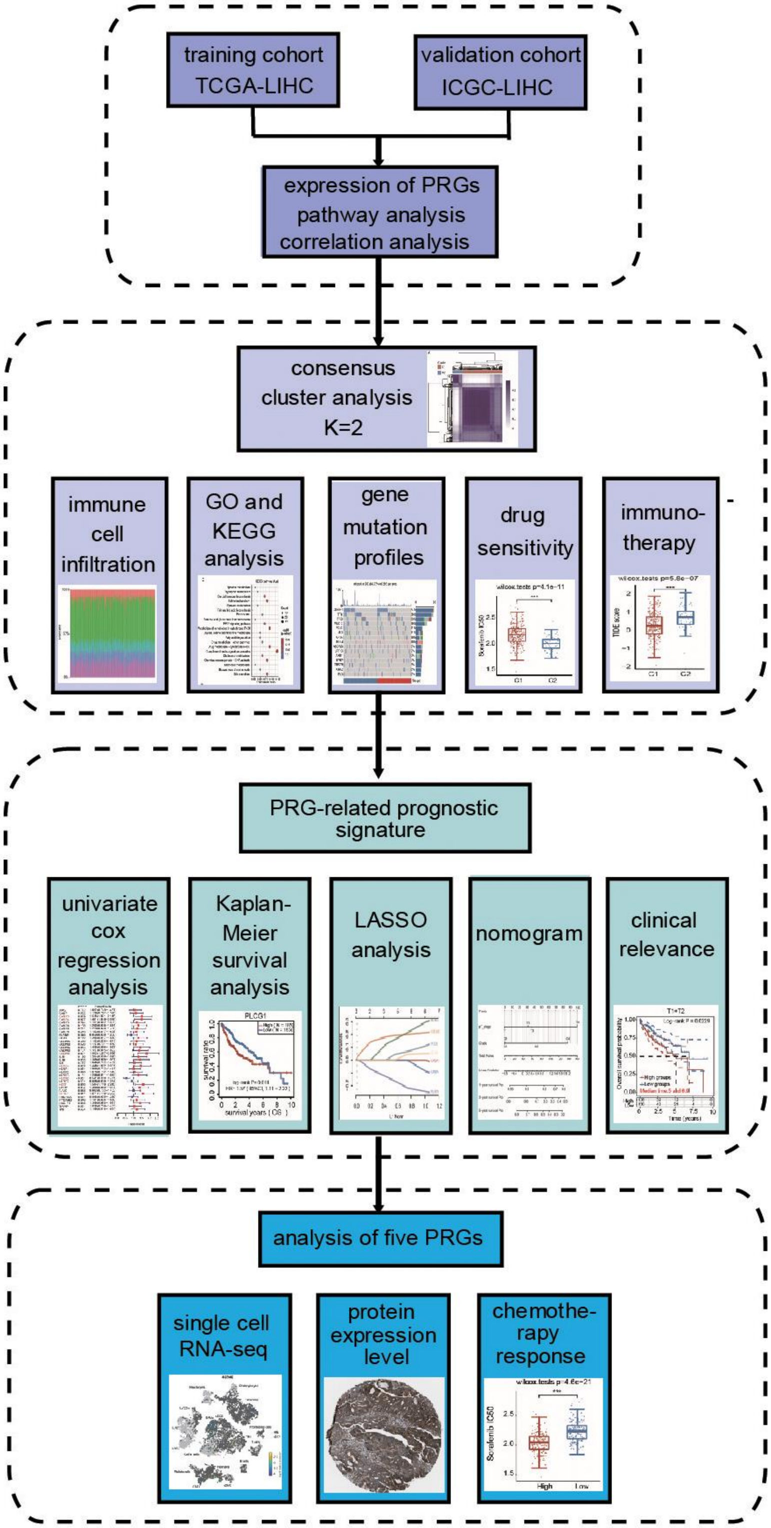

Supplementary Figure 1. The analytical workflow to construct the pyroptosis-related risk model in HCC.

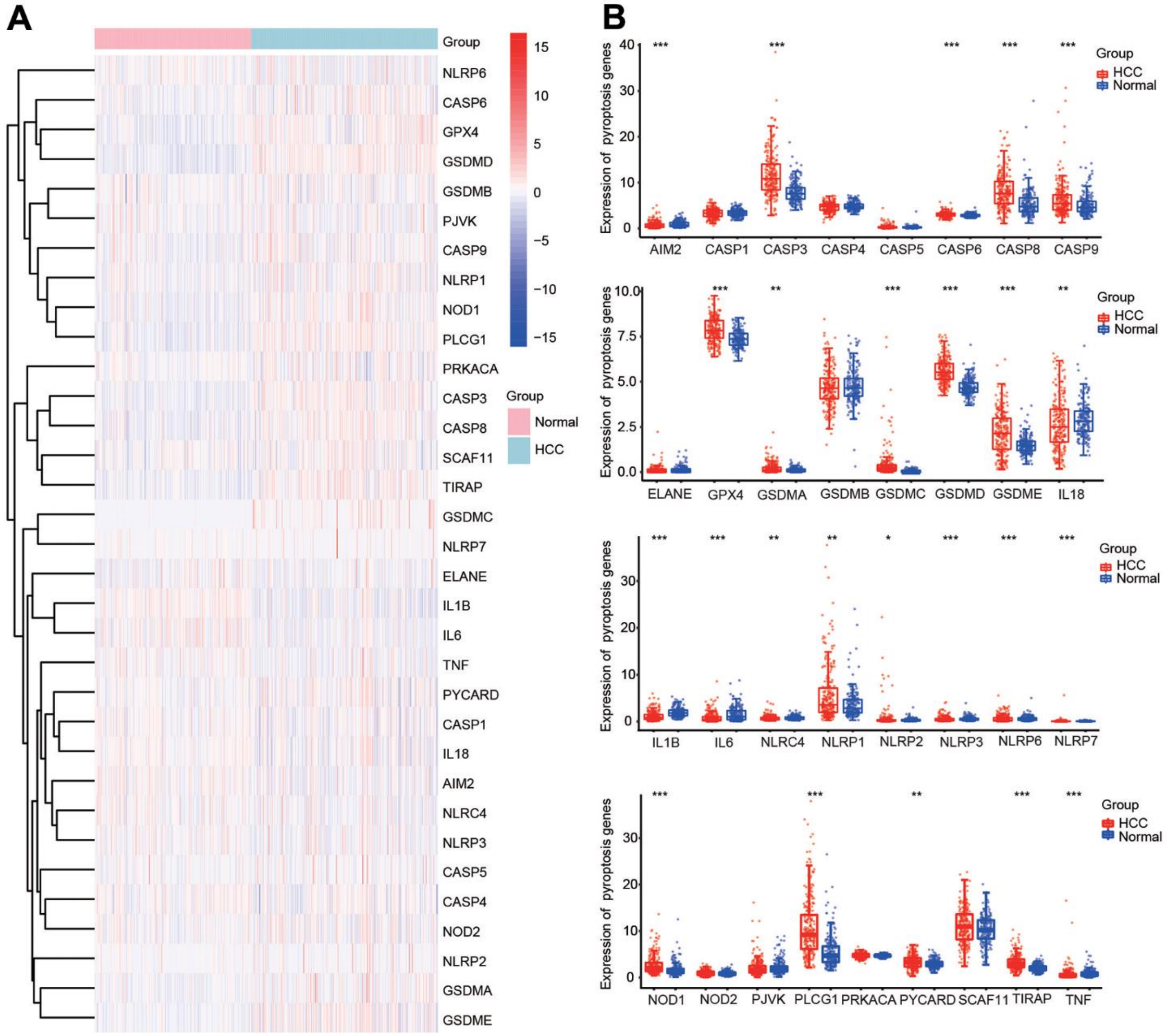

Supplementary Figure 2. Differential expression of 33 PRGs in HCC according to the ICGC database. (**A**) Heatmap of the differential expression of PRGs in HCC samples and normal samples. (**B**) Box diagram of the differential expression of PRGs in HCC samples and normal samples. *p < 0.05, **p < 0.01, ***p < 0.001.

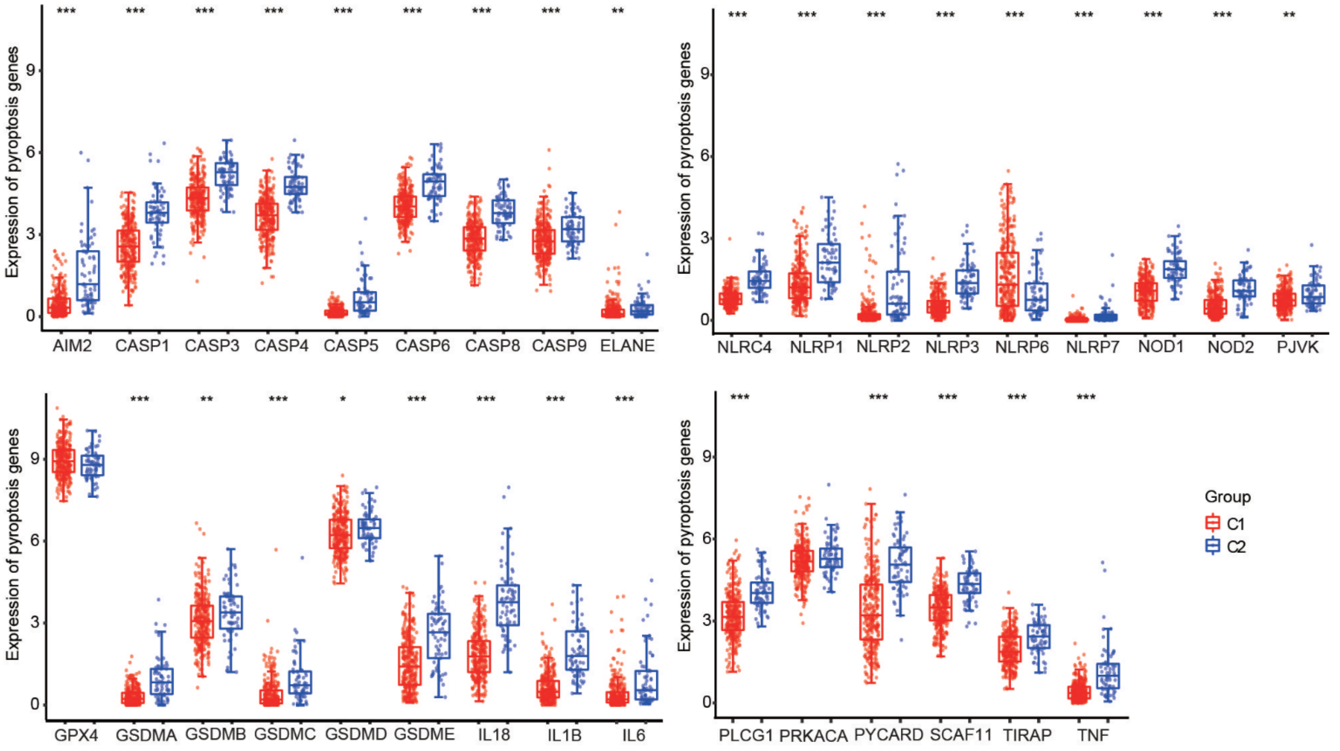

Supplementary Figure 3. Differential expression of 33 PRGs in C1 and C2. *p < 0.05, **p < 0.01, ***p < 0.001.

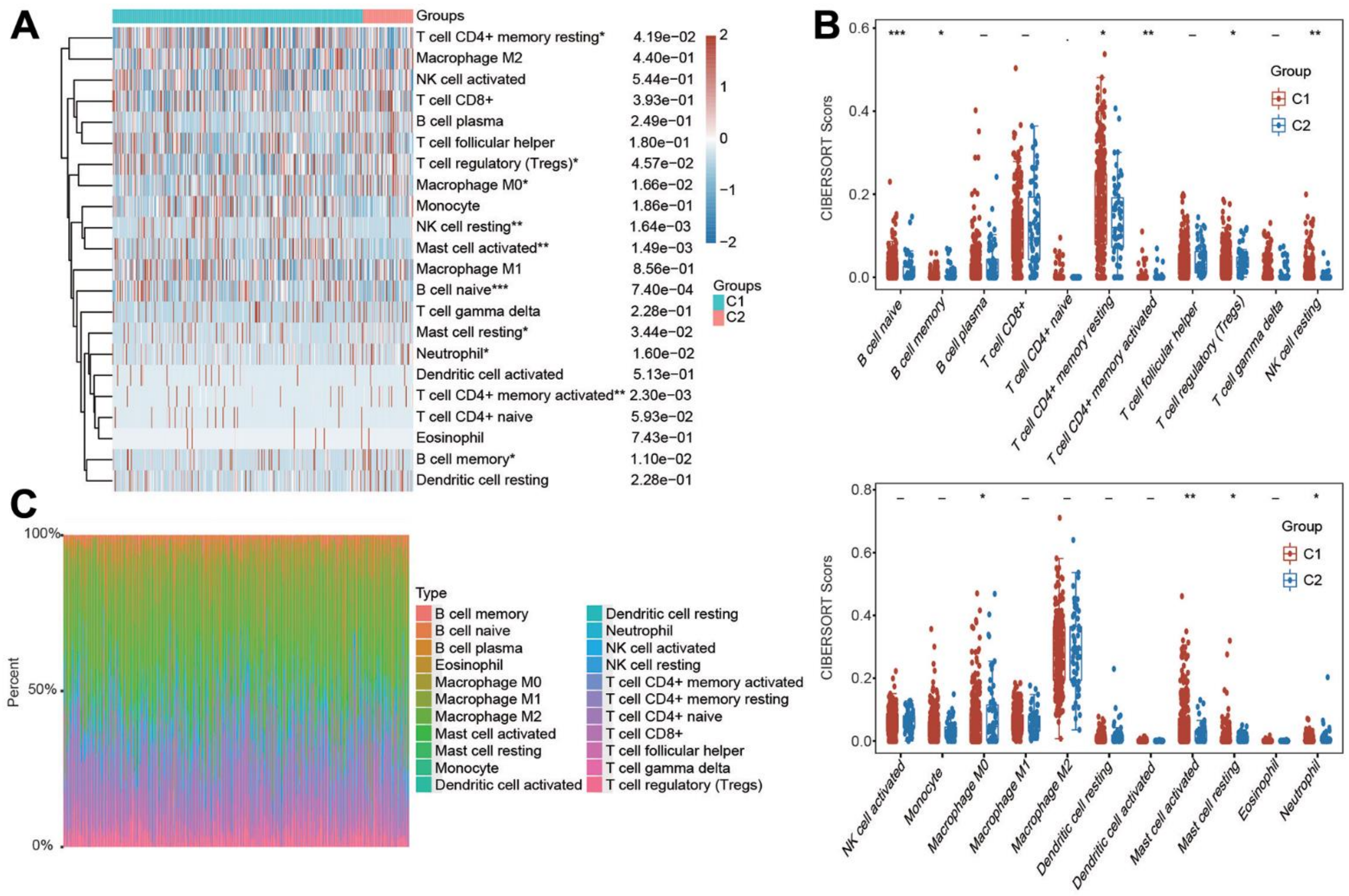

Supplementary Figure 4. Evaluation of immune cell infiltration abundance in different clusters of HCC samples by the CIBERSORT algorithm. (**A**, **B**) Heatmap and box diagram demonstrating the differential infiltration abundance of various immune cells in C1 and C2. (**C**) Bar plot demonstrating the composition of a great variety of immune cells in every HCC patient from the two clusters analyzed by the CIBERSORT algorithm. *p < 0.05, **p < 0.01, ***p < 0.001.

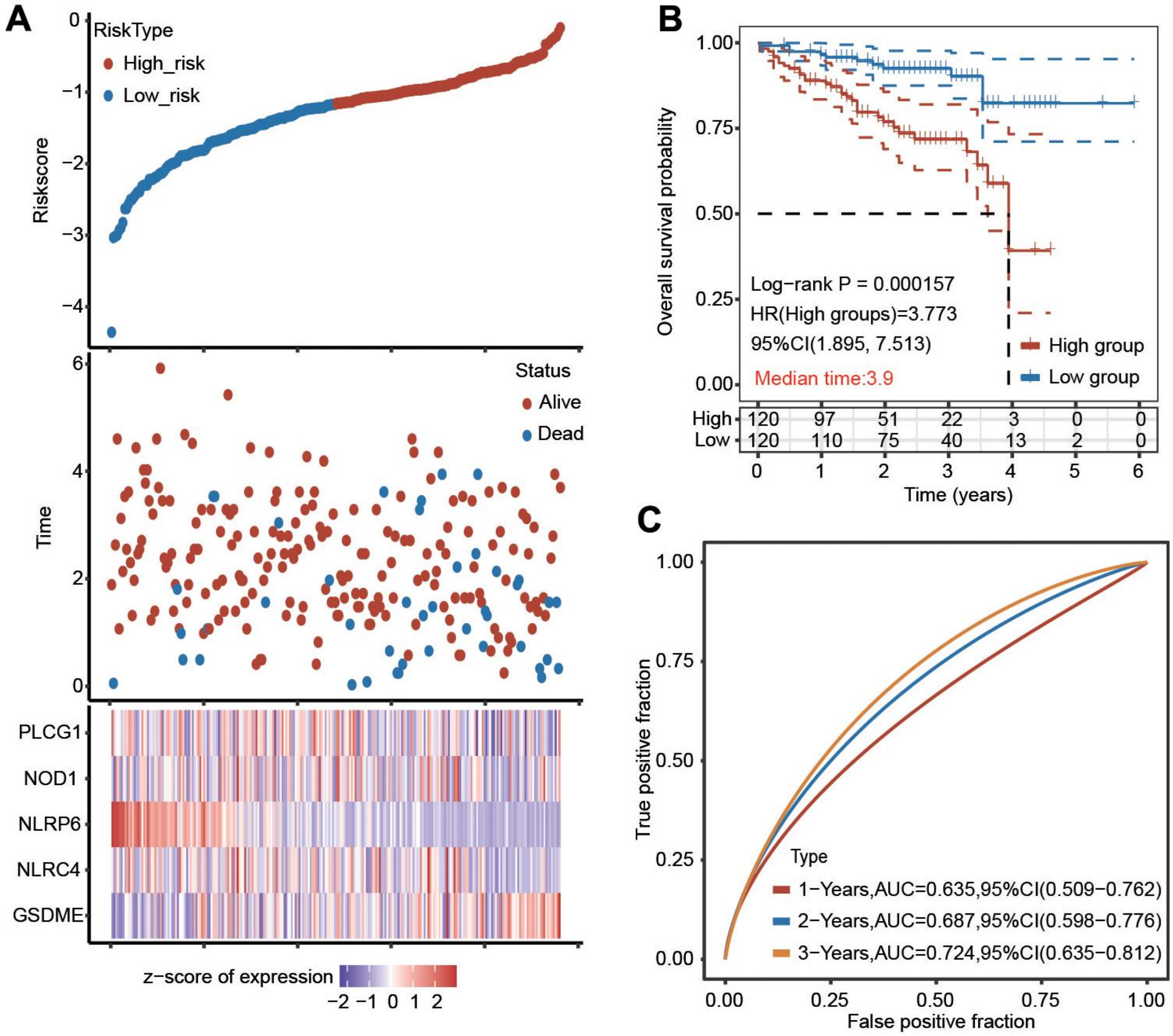

Supplementary Figure 5. Validation of the five-PRG signature model with multivariate Cox regression analysis in the ICGC cohort. (**A**) The risk score and OS status of each case. (**B**) KM analysis for the OS between two subgroups in the ICGC database. (**C**) The AUC of time-dependent ROC curves is shown.

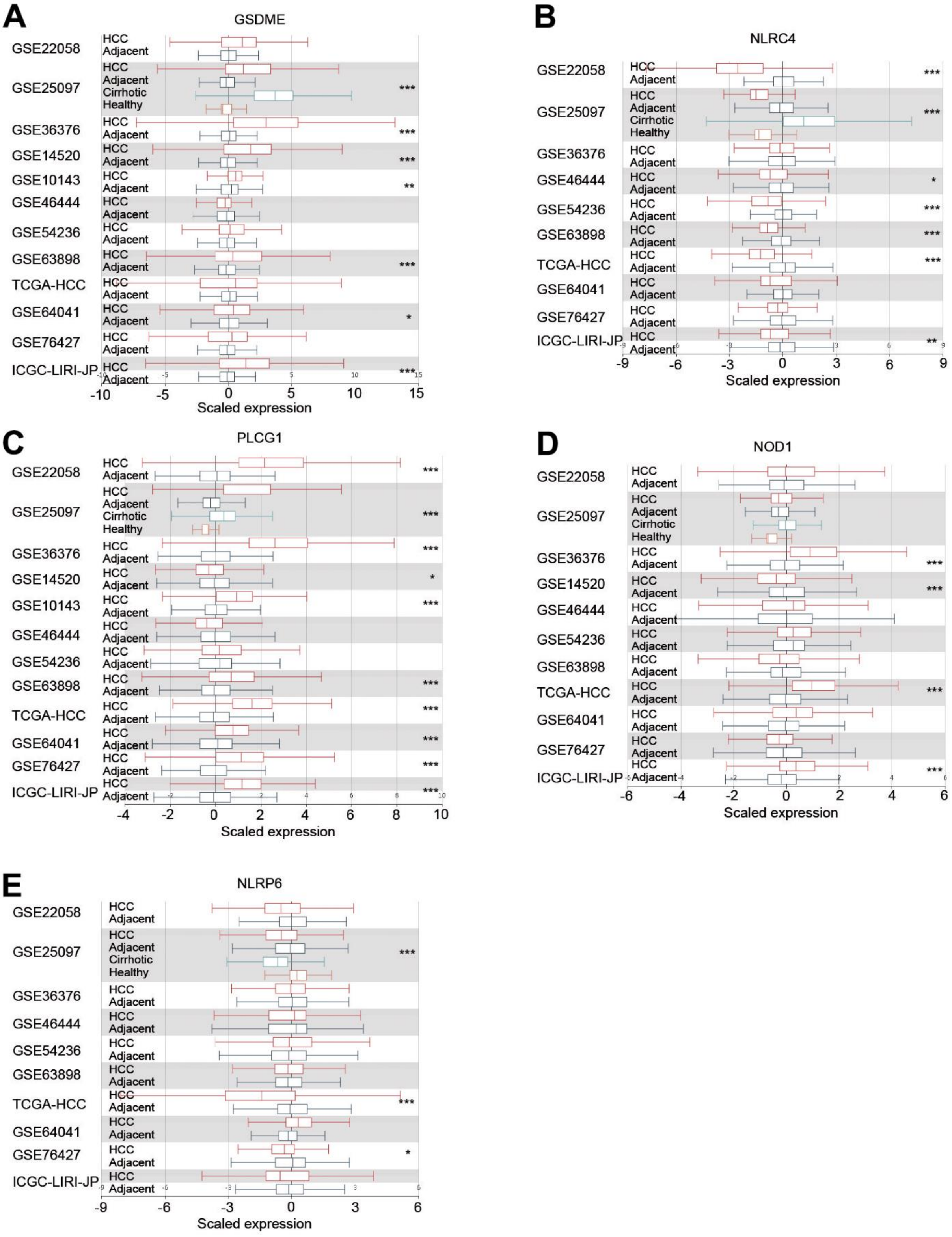

Supplementary Figure 6. Expression of the PRGs. (**A**–**E**) Expression of the 5 PRGs in HCC according to the HCCDB (Integrative Molecular Database of Hepatocellular Carcinoma) database. *p < 0.05, **p < 0.01, ***p < 0.001.

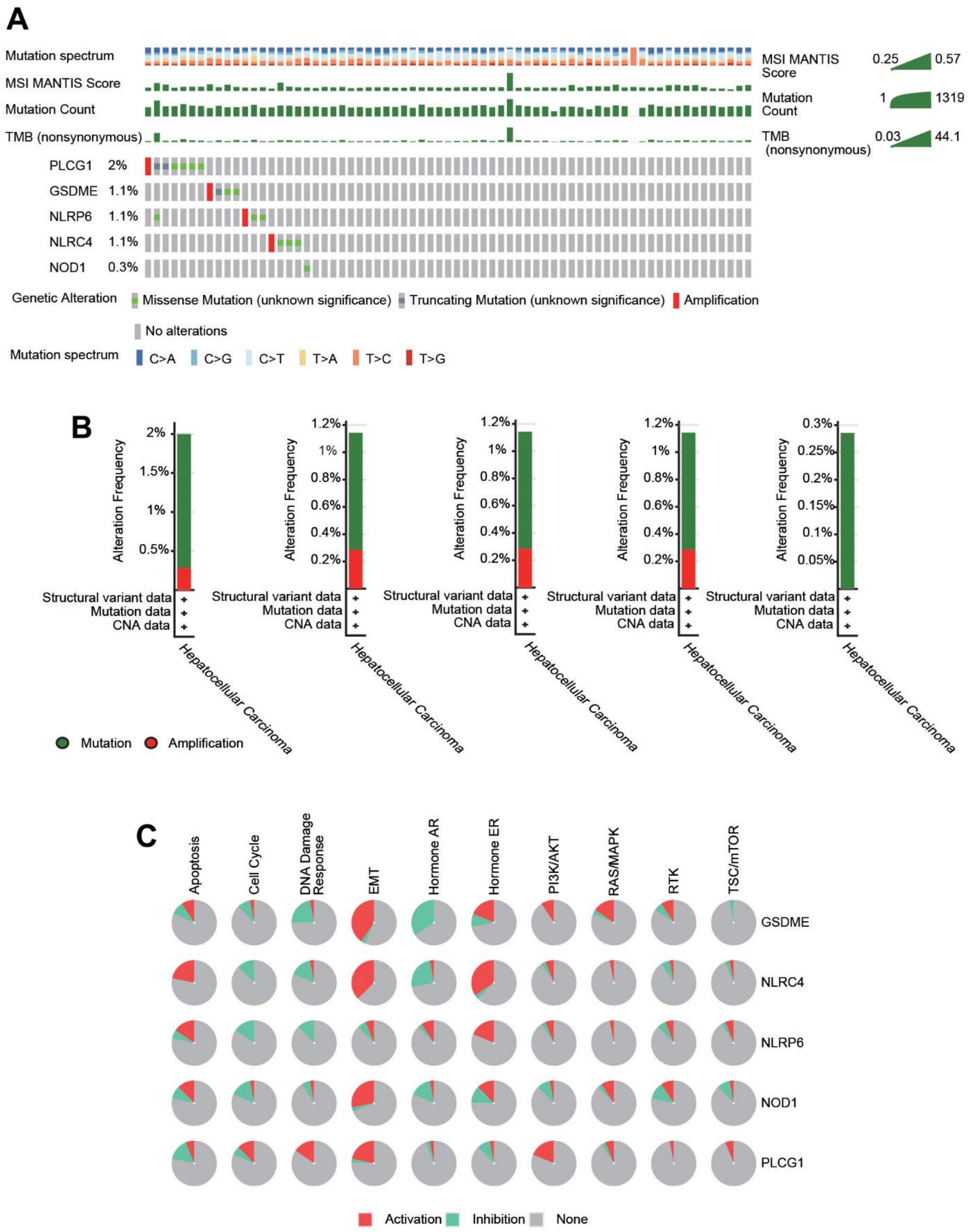

Supplementary Figure 7. Landscape of the genetic alterations of the 5 PRGs and correlation with various signaling pathways. (**A**, **B**) The genetic alterations of PLCG1, GSDME, NLRP6, NLRC4 and NOD1 in HCC were explored using the cBioPortal online tool. (**C**) The relationship between 5 PRGs and multiple cancer-associated signaling pathways was investigated using the GSCALite database.
